# Should a viral genome stay in the host cell or leave? A quantitative dynamics study of how hepatitis C virus deals with this dilemma

**DOI:** 10.1371/journal.pbio.3000562

**Published:** 2020-07-30

**Authors:** Shoya Iwanami, Kosaku Kitagawa, Hirofumi Ohashi, Yusuke Asai, Kaho Shionoya, Wakana Saso, Kazane Nishioka, Hisashi Inaba, Shinji Nakaoka, Takaji Wakita, Odo Diekmann, Shingo Iwami, Koichi Watashi

**Affiliations:** 1 Graduate School of Systems Life Sciences, Kyushu University, Fukuoka, Japan; 2 Department of Virology II, National Institute of Infectious Diseases, Tokyo, Japan; 3 Department of Applied Biological Science, Tokyo University of Science, Noda, Japan; 4 Disease Control and Prevention Center, National Center for Global Health and Medicine, Tokyo, Japan; 5 The Institute of Medical Science, The University of Tokyo, Tokyo, Japan; 6 Graduate School of Mathematical Sciences, The University of Tokyo, Tokyo, Japan; 7 Faculty of Advanced Life Science, Hokkaido University, Sapporo, Japan; 8 PRESTO, Japan Science and Technology Agency, Saitama, Japan; 9 Mathematisch Institute, Universiteit Utrecht, Utrecht, the Netherlands; 10 Department of Biology, Faculty of Sciences, Kyushu University, Fukuoka, Japan; 11 MIRAI, JST, Saitama, Japan; 12 Institute for the Advanced Study of Human Biology (ASHBi), Kyoto University, Kyoto, Japan; 13 NEXT-Ganken Program, Japanese Foundation for Cancer Research (JFCR), Tokyo, Japan; 14 Science Groove, Inc., Fukuoka, Japan; 15 Institute for Frontier Life and Medical Sciences, Kyoto University, Kyoto, Japan; University of Wisconsin-Madison, UNITED STATES

## Abstract

Virus proliferation involves gene replication inside infected cells and transmission to new target cells. Once positive-strand RNA virus has infected a cell, the viral genome serves as a template for copying (“stay-strategy”) or is packaged into a progeny virion that will be released extracellularly (“leave-strategy”). The balance between genome replication and virion release determines virus production and transmission efficacy. The ensuing trade-off has not yet been well characterized. In this study, we use hepatitis C virus (HCV) as a model system to study the balance of the two strategies. Combining viral infection cell culture assays with mathematical modeling, we characterize the dynamics of two different HCV strains (JFH-1, a clinical isolate, and Jc1-n, a laboratory strain), which have different viral release characteristics. We found that 0.63% and 1.70% of JFH-1 and Jc1-n intracellular viral RNAs, respectively, are used for producing and releasing progeny virions. Analysis of the Malthusian parameter of the HCV genome (i.e., initial proliferation rate) and the number of de novo infections (i.e., initial transmissibility) suggests that the leave-strategy provides a higher level of initial transmission for Jc1-n, whereas, in contrast, the stay-strategy provides a higher initial proliferation rate for JFH-1. Thus, theoretical-experimental analysis of viral dynamics enables us to better understand the proliferation strategies of viruses, which contributes to the efficient control of virus transmission. Ours is the first study to analyze the stay-leave trade-off during the viral life cycle and the significance of the replication-release switching mechanism for viral proliferation.

## Abstract 2

A theoretical-experimental analysis of viral dynamics reveals a stay/leave trade-off during the viral life cycle and demonstrates the significance for viral proliferation of a replication-release switching mechanism.

## Introduction

**Fig 1:**
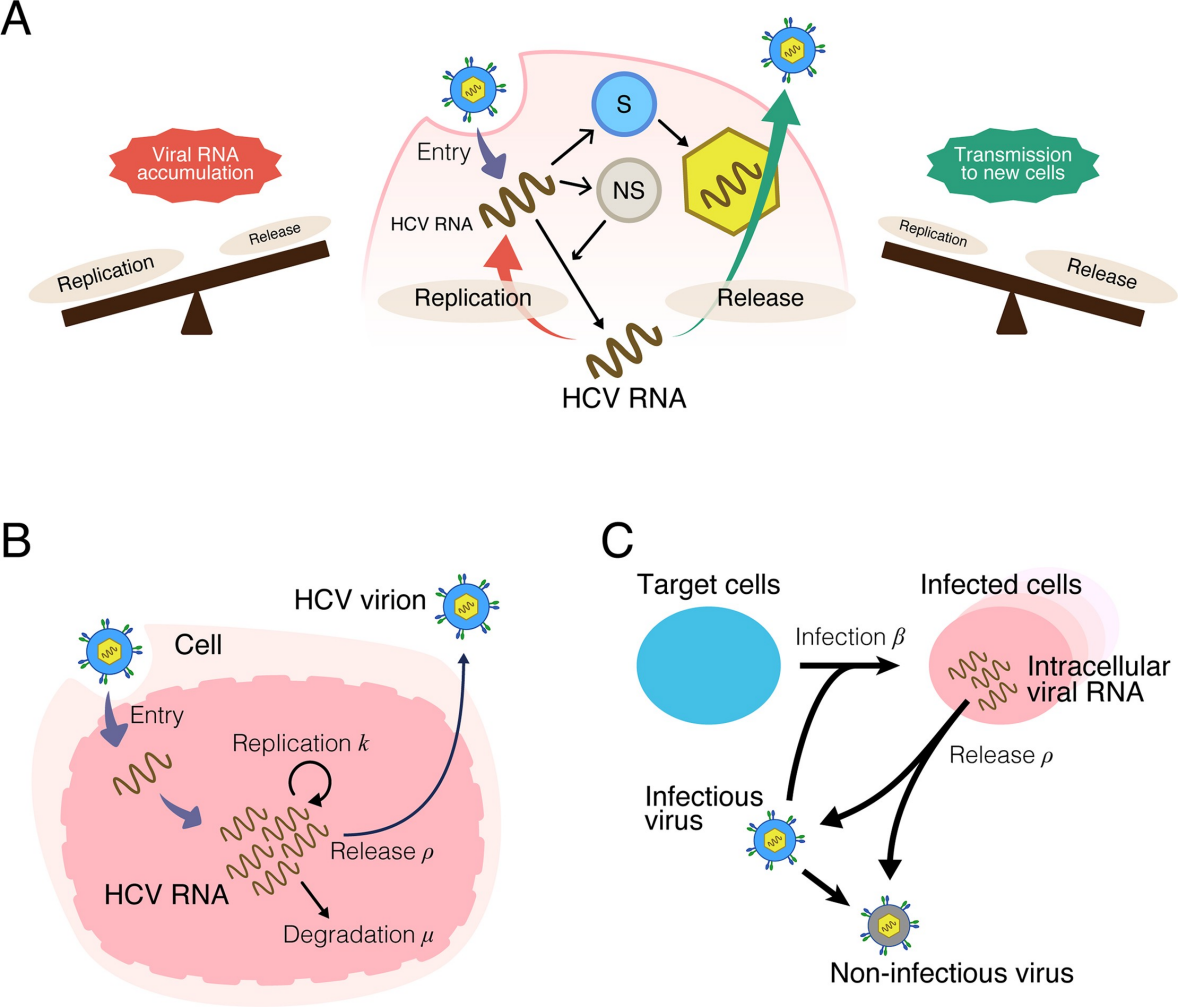
Schematic representation of multiscale HCV infection and mathematical model. **(A)** Schematic representation of intracellular HCV life cycle and trade-off between viral replication and release of intracellular viral RNA. Viral RNA in cells is translated to produce S and NS proteins. Viral RNA is either amplified through the functions of NS proteins through replication or is assembled with S proteins and released as a progeny virus. If the balance between viral replication and release leans toward replication, intracellular viral RNAs will accumulate. In contrast, high rates of intracellular RNA release will create opportunities for transmission to new cells but will deplete viral RNA in the cell. **(B)** Modeling the intracellular virus life cycle. Intracellular viral RNA either replicates inside the cell at rate *k*, is degraded at rate *μ*, or assembles with viral proteins to be released within HCV virions at rate *ρ*. **(C)** Multiscale modeling of intracellular replication and intercellular infection. Target cells are infected by infectious viruses at rate *β*. HCV, hepatitis C virus; NS protein, nonstructural protein; S protein, structural protein.

Hepatitis C virus (HCV) is an RNA virus specifically infecting liver cells. HCV produces progeny viruses rapidly, with approximately 10^12^ copies sometimes observed in patients [1]. Following virus entry into target cells, viral genomic RNA produces structural proteins (S proteins) and nonstructural proteins (NS proteins) (Fig 1A). Using the genomic RNA as a template, the viral NS proteins amplify HCV RNA (“RNA replication”). Genomic RNA can also be assembled with viral S proteins into progeny virions to be egressed outside of cells, creating the opportunity for transmission (in this study, we call the process including particle assembly and egress “release”). Thus, a single HCV genomic RNA molecule can be used either for RNA replication or for release, and the balance between these processes governs viral proliferation. The molecular mechanisms underlying each event in the viral life cycle have been extensively investigated [2,3], yet the replication-release trade-off and its significance for viral proliferation remain poorly understood.

HCV JFH-1 is a genotype 2a strain isolated by our group from a patient with fulminant hepatitis [4]. JFH-1 has been a standard strain used for experiments to characterize HCV infection, virus-host interactions, and immune responses against HCV [4]. In addition, Jc1 or J6/JFH (a chimeric strain in which a region of the JFH-1 genome from the core to NS2 was replaced by sequences from another genotype 2a virus, the J6 strain) was developed as a laboratory strain to improve virus production. Jc1 is used for development of antiviral agents and vaccines, which requires large amounts of virus [5,6]. In spite of their high sequence similarity (97% identity over the whole genome), these two viruses have different virological characteristics especially in terms of the release process: whereas JFH-1 particles assemble on lipid droplet membranes, particle assembly of J6/JFH-1-chimeric laboratory strains is associated with endoplasmic reticulum–derived membranes [2,3]. Thus, these two related strains are a useful reference set to compare the dynamics of release and RNA replication.

In this study, we used a cell culture model of infection with these two HCV reference strains and measured the time course of viral production (including HCV RNA inside cells and virions produced outside of the cells), infectivity of progeny HCV, and infected cell numbers. We also developed a multiscale mathematical model to describe intra- and intercellular HCV dynamics. This interdisciplinary approach suggests that different strategies exist for viral proliferation: the stay-at-home strategy (JFH-1) and the leaving-home strategy (Jc1-n, a J6/JFH-1-chimeric strain). We discuss the relevance of these strategies for viral proliferation, while referring to [7] for wider evolutionary context.

## Results

### Age-structured multiscale modeling of HCV infection

To describe the intracellular replication dynamics of HCV viral RNA, we used the following mathematical model:

$$\frac{dR(a)}{da}=kR(a)-(\mu +\rho )R(a),$$ (1)

where *R*(*a*) is the amount of intracellular viral RNA in cells that have been infected for time *a*. The intracellular viral RNA replicates at rate *k* per day, degrades at rate *μ* per day, and is released to extracellular space at rate *ρ* per day (Fig 1B). Note that if viruses have small or large *ρ*, then they tend to stay inside or leave the cell, respectively (see later). In our virus experiments (see Methods), the released viruses could infect other target cells. To describe multiround virus transmission (i.e., de novo infection), we needed to couple intracellular viral replication with a standard mathematical model for intercellular virus infection in cell culture [8,9] (Fig 1C). In S1 Text, we derived the following multiscale ordinary differential equation (ODE) model for HCV infection from the corresponding age-structured partial differential equation (PDE) model [10,11]:

$$\frac{dT(t)}{dt}=gT(t)\left(1-\frac{T(t)+I(t)}{K}\right)-\beta_{\theta }T(t)V_{\theta }(t),$$ (2)

$$\frac{dI(t)}{dt}=gI(t)\left(1-\frac{T(t)+I(t)}{K}\right)+\beta_{\theta }T(t)V_{\theta }(t),$$ (3)

$$\frac{dA(t)}{dt}=\beta_{\theta }T(t)V_{\theta }(t)+(k-\mu -\rho )A(t),$$ (4)

$$\frac{dV_{\theta }(t)}{dt}=f_{\theta }\rho A(t)-rV_{\theta }(t)-cV_{\theta }(t),$$ (5)

$$\frac{dV(t)}{dt}=\rho A(t)-cV(t).$$ (6)

Here, the intercellular variables *T*(*t*) and *I*(*t*) represent the numbers of uninfected and infected target cells (cells/well), respectively, and *V*(*t*) and *V*_*θ*_(*t*) represent the total amount of extracellular viral RNA (copies/well) and the extracellular viral infectious titer expressed (focus formation unit [ffu]/well), respectively. The intracellular variable *A*(*t*) represents the total amount of intracellular viral RNA. The parameters *g* and *K* represent the growth rate (per day) and the carrying capacity of target cells, respectively, and *β*_*θ*_ and *f*_*θ*_ are the converted infection rate constant ([ffu/well・day]^-1^) and the fraction of infectious virus (RNA copies・ffu^-1^), respectively. Note that we assume no cellular loss induced by infection because HCV is a noncytopathic virus [4]. We assumed that progeny viruses were cleared at rate *c* per day via degradation at rate *c*_*RNA*_ per day and washing at rate *c*_*w*_ per day (i.e., *c* = *c*_*RNA*_+*c*_*w*_), and that infectious virions lose infectivity at rate *r*. Because of the different RNA sequence and the resulting different RNA modification status as well as the different physical property of virions, specific stability/degradation rate of each virus strain is expected. Separately, we directly estimated *g*, *K*, *c*, *μ*, and *r* for both HCV JFH-1 and Jc1-n in S1 Fig. Detailed explanations of Eqs 2–6 are given in S1 Text.

**Fig 2:**
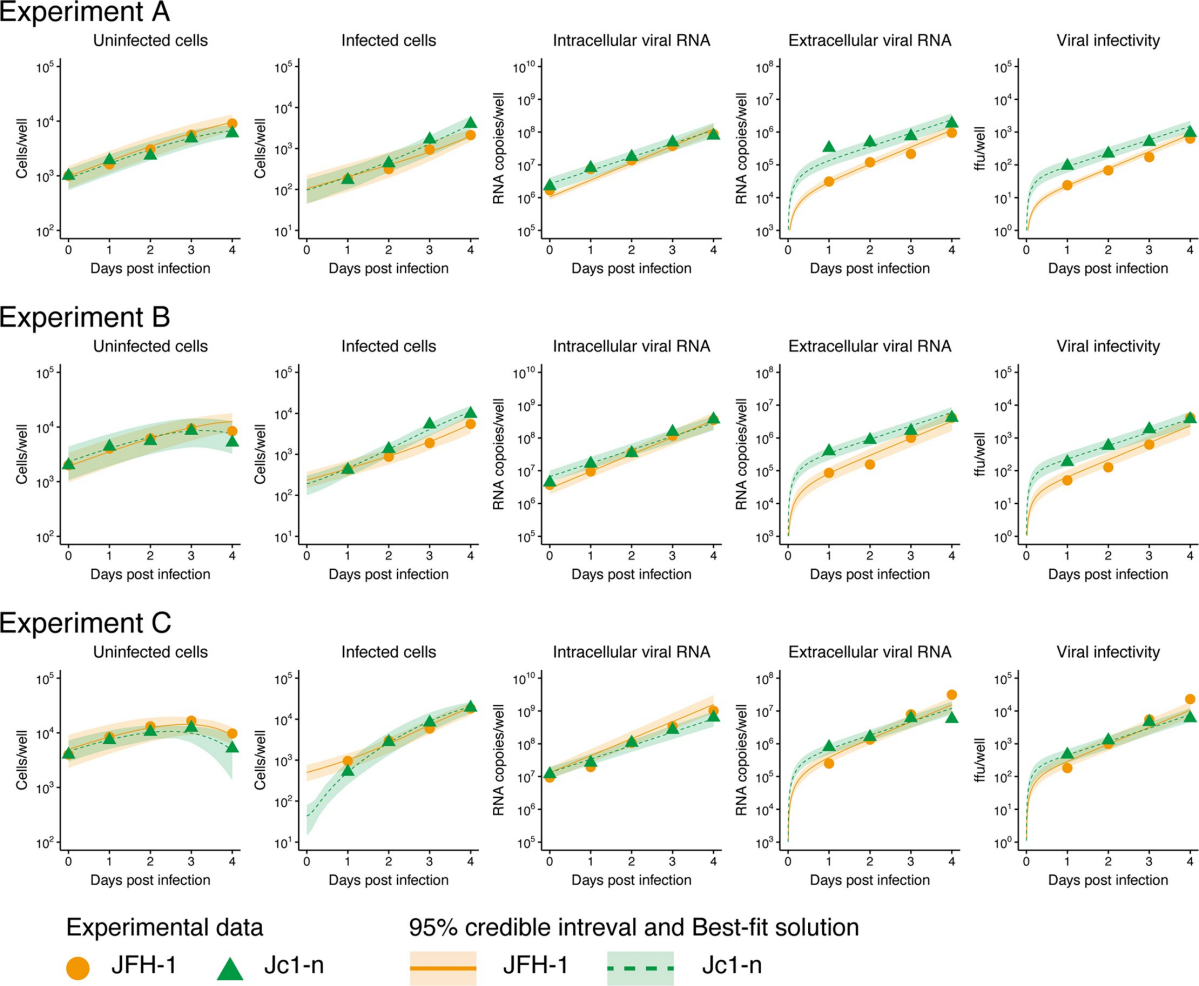
Dynamics of HCV JFH-1 and Jc1-n infection in cell culture. Fitting of the mathematical model to the experimental data of HCV JFH-1 and Jc1-n infection in cell culture. Three different numbers of Huh-7 cells infected with either HCV JFH-1 or Jc1-n 1 day after inoculation were seeded (experiment A: 1,000, experiment B: 2,000, and experiment C: 4,000 cells per well) and chased to detect the following values at days 0, 1, 2, 3, and 4 post seeding (log_10_ scale): numbers of uninfected and infected cells, amount of intracellular and extracellular viral RNA (copies/well), and extracellular viral infectivity (ffu/well) (orange circle: JFH-1, green triangle: Jc1-n). The shadowed regions correspond to 95% posterior intervals and the solid curves give the best-fit solution (mean) for Eqs 2–6 to the time-course dataset. All data for each strain were fitted simultaneously. The underlying data for this figure can be found in S1 Data. ffu, focus formation unit.

To assess the variability of kinetic parameters and model predictions, we performed Bayesian estimation for the whole dataset using Markov chain Monte Carlo (MCMC) sampling (see Methods). Simultaneously, we fitted Eqs 2–6 to the experimentally determined numbers of uninfected and infected cells (cells/well), extracellular viral RNA (copies/well) and infectious titer (ffu/well), and intracellular viral RNA (copies/well). These figures were derived from infection experiments using different numbers of plated cells for either HCV JFH-1 or Jc1-n as described previously [8,9,12,13]. The remaining free model parameters (i.e., *β*_*θ*_, *k*, *ρ*, *f*_*θ*_) along with initial values for variables (i.e., *T*(0), *I*(0), *A*(0), *V*_*θ*_(*t*), *V*(0)) were estimated from the MCMC sampling. Experimental measurements below the detection limit were excluded in the fitting. The estimated parameters and initial values are listed in Table 1 and S1 Table. The typical behavior of the model using these best-fit parameter estimates is shown together with the data in Fig 2 for HCV JFH-1 (orange) and Jc1-n (green) (see Methods for HCV strains) and indicated that Eqs 2–6 described the in vitro data very well. The shadowed regions corresponded to 95% posterior predictive intervals, the solid and dashed lines gave the best-fit solution (mean) for Eqs 2–6, and the orange circles and green triangles showed the experimental datasets.

**Table 1:** Parameter values estimated from the cell culture infection experiment.

Parameter Name	Symbol	Unit	HCV JFH-1		HCV Jc1	
			Value	95% CI	Value	95% CI
Fitted parameters from separate experiments						
Rate of virion infectivity loss	*r*	day^-1^	1.52	―	2.16	―
Degradation rate of extracellular viral RNA	*c*_*RNA*_	day^-1^	7.55×10^−2^	(0.238–1.99)×10^−1^	0.240	0.238–0.469
Clearance rate of extracellular viral RNA due to washing	*c*_*w*_	day^-1^	1.18	―	1.82	―
Degradation rate of intracellular viral RNA	*μ*	day^-1^	2.54	2.14–3.01	2.75	2.21–3.43
Estimated parameters from in vitro total cell growth data						
Proliferation rate of Huh-7 cells	*g*	day^-1^	0.670	―	0.665	―
Carrying capacity of Huh-7 cells	*K*	cells	4.12×10^4^	―	3.75×10^4^	―
Parameters obtained from simultaneous fitting of full in vitro dataset						
Rate constant for infections	*β*_*θ*_	(ffu/well・day)^-1^	1.26×10^−4^	(0.788–1.89)×10^−4^	2.29×10^−4^	(1.32–3.64)×10^−4^
Replication rate of intracellular viral RNA	*k*	day^-1^	3.75	3.64–3.86	3.75	3.64–3.86
Release rate of intracellular viral RNA	*ρ*	day^-1^	2.36×10^−2^	(1.73–3.15)×10^−2^	6.38×10^−2^	(4.20–9.34)×10^−2^
Converted fraction of infectious viral RNA	*f*_*θ*_	RNA copies・ffu^-1^	1.19×10^−3^	(0.970–1.44)×10^−3^	1.08×10^−3^	(0.773–1.48)×10^−3^

Abbreviations: ffu, focus formation unit; HCV, hepatitis C virus

Using the estimated parameters shared between the original PDE model in S1 Text and the transformed ODE model (i.e., Eqs 2–6), we successfully reconstructed age information for intracellular viral RNA in infected cells of infection age *a*, which cannot be obtained through conventional experiments alone (S2A Fig). S2B Fig shows the differences in intracellular JFH-1 and Jc1-n viral RNA levels in cells of infection age *a*. At the beginning of the experiment, intracellular viral RNA increased faster under Jc1-n infection than under JFH-1 infection (shown in green). However, intracellular JFH-1 viral RNA gradually accumulated to higher levels than Jc1-n at later time points after infection (shown in yellow to brown). These data illustrated the different dynamics of these two strains and the impact of these dynamics on intracellular viral RNA production, all resulting from different strategies to transmit the viral genome (see below).

### Dynamics of HCV JFH-1 and Jc1-n strain replication

**Fig 3:**
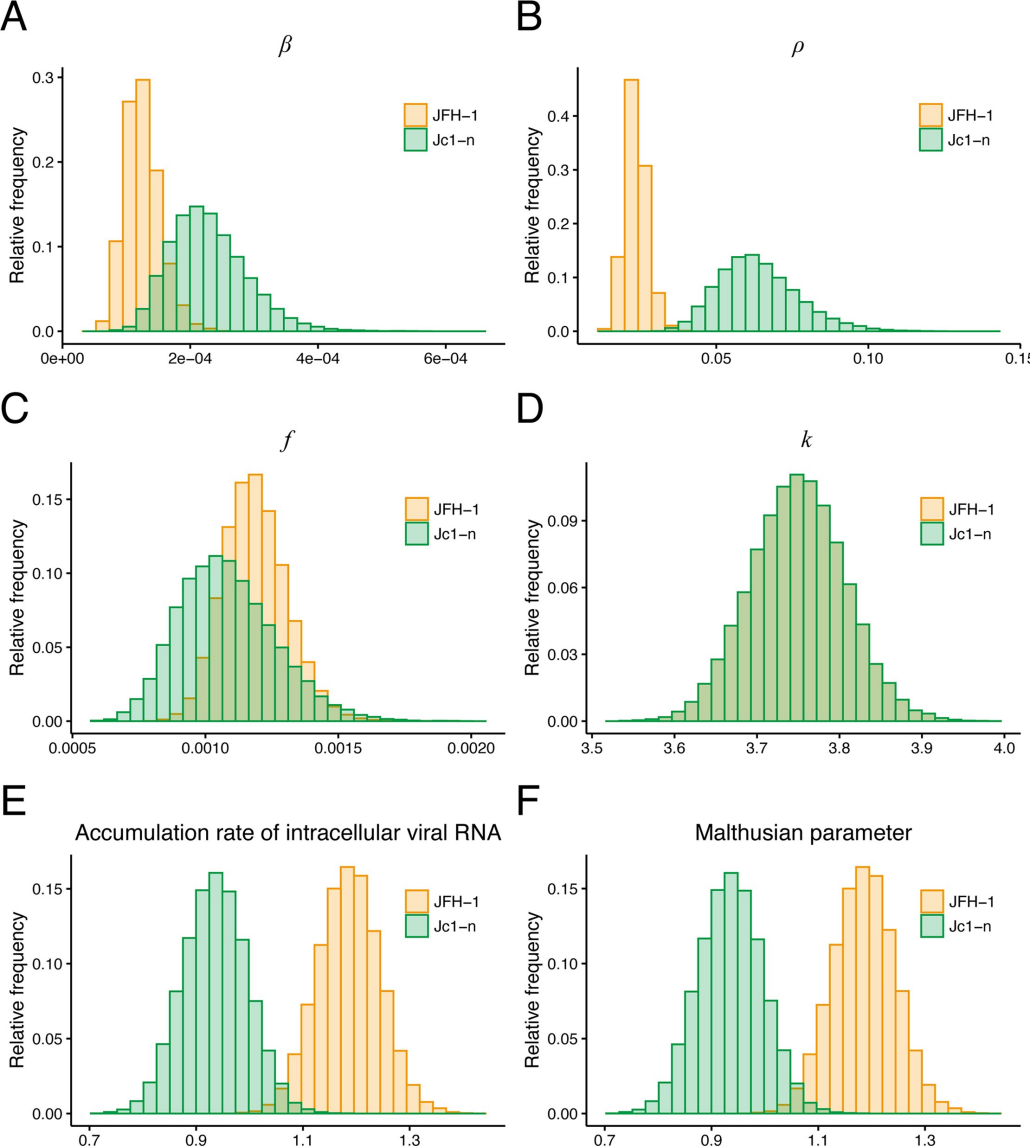
Characterization of viral dynamics of HCV JFH-1 and Jc1-n. The distributions of the rate constant for infection, *β*_*θ*_; the release rate of intracellular viral RNA, *ρ*; the converted fraction of infectious viral RNA, *f*_*θ*_; and the replication rate of intracellular viral RNA, *k*, inferred by MCMC computations are shown in **(A)**, **(B)**, **(C)** and **(D)**, respectively, for HCV JFH-1 (orange) and Jc1-n (green). Parameters *β*_*θ*_ and *ρ* for Jc1-n were significantly larger than for JFH-1, whereas there was no significant difference in *f*_*θ*_ between the two strains as assessed by repeated bootstrap *t* test. JFH-1 and Jc1-n stains had identical viral RNA replication rates. The distributions of accumulation rates of intracellular viral RNA, *k*−*μ*−*ρ*, and the Malthusian parameter, *M*, calculated from all accepted MCMC parameter estimates are shown in **(E)** and **(F)**, respectively, for HCV JFH-1 (orange) and Jc1-n (green). These indices were significantly larger for JFH-1 than for Jc1-n as assessed by the repeated bootstrap *t* test. The underlying data for this Figure can be found in S2 Data. HCV, hepatitis C virus; MCMC, Markov chain Monte Carlo.

Our model (Eqs 2–6) applied to time-course experimental data allowed us to extract the following kinetic parameters: the distribution of the rate constant for infection, *β*_*θ*_; the release rate of intracellular viral RNA, *ρ*; the converted fraction of infectious viral RNA, *f*_*θ*_; and the replication rate of intracellular viral RNA, *k* (Fig 3 and Table 1). Comparing these parameters for JFH-1 and Jc1-n showed a significant difference between the rate constant for infections, *β*_*θ*_, of JFH-1 (1.26×10^−4^ [ffu/well・day]^-1^, 95% CI 0.788–1.89×10^−4^ [ffu/well・day]^-1^) and Jc1-n (2.29×10^−4^ [ffu/well・day]^-1^, 95% CI 1.32–3.64×10^−4^ [ffu/well・day]^-1^) (*p* = 1.48×10^−4^ by repeated bootstrap *t* test) (Fig 3A). In addition, the release rates of intracellular viral RNA, *ρ*, for JFH-1 and Jc1-n were 2.36×10^−2^ per day (95% CI 1.73–3.15×10^−2^) and 6.38×10^−2^ per day (95% CI 4.20–9.34×10^−2^), respectively (*p*<0.01 by repeated bootstrap *t* test) (Fig 3B). These estimates indicated that Jc1-n infects cells 1.82 times faster and produces progeny viruses from infected cells 2.70 times faster than JFH-1. The estimate was further validated by independent experiments, in which Jc1-n entry and virus production were indeed significantly higher than those of JFH-1, although early replication was similar for the two strains (S2 Text and S3A–S3C Fig). The cell line used in our in vitro model was deficient in interferon induction by HCV infection (S3D Fig), as reported previously [14]. No significant difference was apparent in the converted fraction of infectious virus, *f*_*θ*_ (Fig 3C). Because JFH-1 and Jc1-n have identical nonstructural regions essential for RNA replication (NS3–NS5B), we estimated the same viral RNA replication rate, *k*, for these two viruses (Fig 3D). Hence, our parameter estimation captured the characteristics of the two strains well and was able to quantitatively describe viral infection dynamics.

In our multiscale model (Eqs 2–6), the accumulation rate of intracellular viral RNA was defined as the difference between the replication rate and the sum of the degradation rate and the release rate (i.e., *k*−*μ*−*ρ*). The distributions of calculated intracellular RNA accumulation rates for JFH-1 (1.19 per day, 95% CI 1.07–1.30) and Jc1-n (0.936 per day, 95% CI 0.819–1.05) are shown in Fig 3E (*p* = 8×10^−6^ by bootstrap *t* test) (Table 1). The preferential accumulation of JFH-1 RNA inside cells was consistent with its tendency toward gradual increased levels of intracellular RNA at later time points (S2B Fig). The difference in the site of virion assembly and the trafficking pathway as well as in the viral genome RNA stability may explain the higher accumulation of JFH-1 RNA in the cells. To further evaluate the total viral RNA level considering multiround virus transmission, the Malthusian parameter, *M*, was used as an indicator of the initial growth rate of intracellular viral RNA for each HCV strain [8,12,15]. Here, the Malthusian parameter was given by

$$M=\frac{k-\mu -\rho -r-c+\sqrt{{\left(k-\mu -\rho +r+c\right)}^{2}+4\beta_{\theta }Kf_{\theta }\rho }}{2}$$ (7)

(see S3 Text for derivation of the Malthusian parameter). The Malthusian parameters for JFH-1 and Jc1-n were calculated as 1.19 (95% CI 1.07–1.30) and 0.936 (95% CI 0.819–1.05), respectively, and were significantly different from one another (*p* = 8×10^−6^ by bootstrap *t* test) (Fig 3F and Table 1). Interestingly, even if Jc1-n had a larger infection rate, *β*_*θ*_, and release rate, *ρ*, compared with JFH-1, the initial growth rate of total JFH-1 RNA was higher than that of Jc1-n. This result demonstrated that the capacity to accumulate viral RNA inside cells predominantly determines the initial growth rate rather than release of progeny viruses.

### Stay-at-home strategy or leaving-home strategy for “optimizing” HCV proliferation

**Fig 4:**
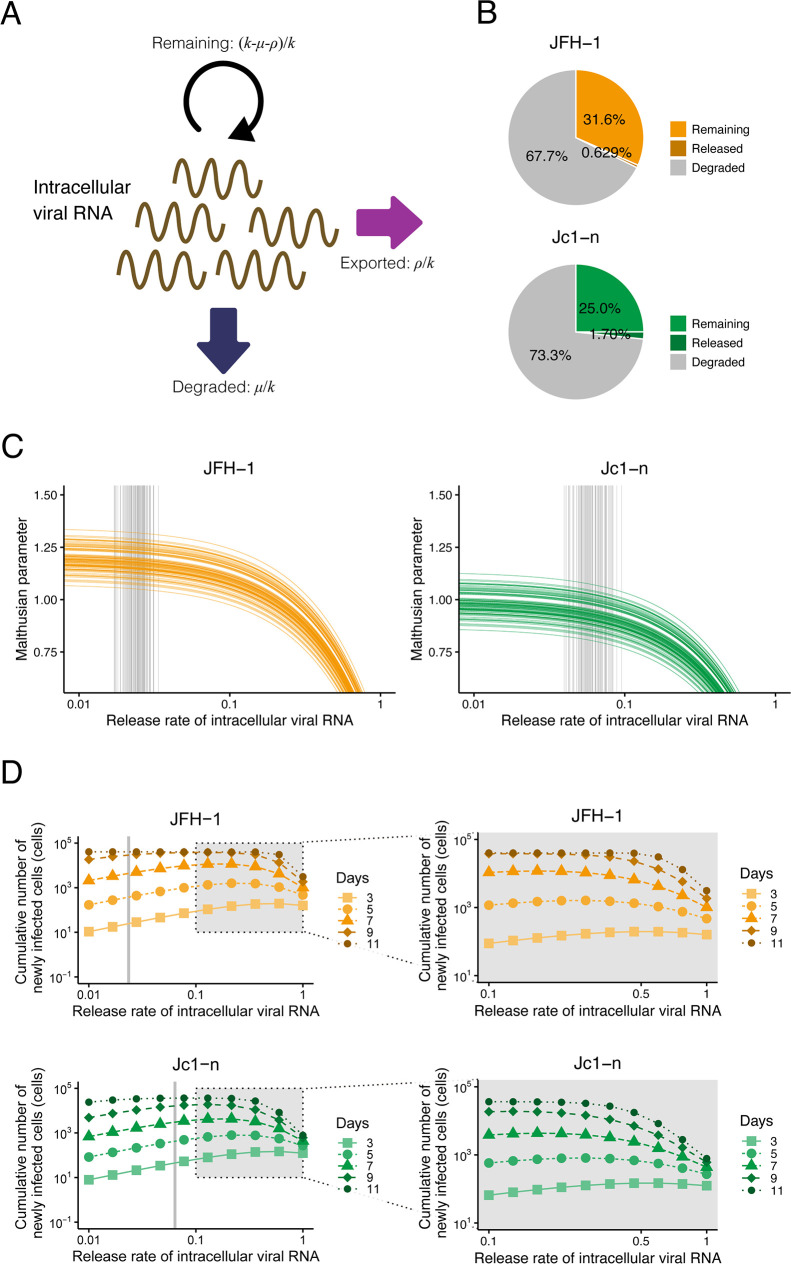
Different strategies adopted by JFH-1 and Jc1-n for viral proliferation. **(A)** Schematic representation of the fate of replicated intracellular viral RNA. Viral RNA is used either for driving RNA replication in cells or for producing progeny viruses for release outside cells or is degraded. **(B)** Percentage of replicated intracellular HCV JFH-1 and Jc1-n viral RNA that remains inside cells, is released outside cells, and is degraded. **(C)** Change in the Malthusian parameter (Eq 7) with various release rates of intracellular viral RNA. The orange and green curves show Malthusian parameters calculated using 100 parameter sets sampled from MCMC parameter estimates as functions of *ρ* for JFH-1 and Jc1-n, respectively. The gray vertical lines are the corresponding release rates estimated from the actual experimental data. **(D)** Change in the cumulative number of newly infected cells with the various release rates (Eq 8). (Left panels) The orange and green curves represent the cumulative numbers of newly infected cells until 3, 5, 7, 9, and 11 days post infection calculated using the means of estimated parameters as a function of *ρ* for JFH-1 and Jc1-n, respectively. The gray vertical line represents the mean release rate estimated from the experimental data. (Right panels) Enlarged views of the gray zones in left panels. Finely calculated cumulative numbers of newly infected cells are shown. The underlying data for this figure can be found in S3 Data. HCV, hepatitis C virus; MCMC, Markov chain Monte Carlo.

We investigated how differences between the two strains, JFH-1 and Jc1-n, might be interpreted in an evolutionary perspective. As mentioned above, we considered two opposing strategies: the “stay-at-home strategy” and the “leaving-home strategy”: if viruses have smaller *ρ*, they preferentially stay inside the cell, but if they have larger *ρ*, they leave the cell. To quantitatively characterize these different strategies, we defined the fraction of viral RNA remaining in the cells ((*k*−*μ*−*ρ*)/*k*), released from the cells (*ρ*/*k*), and degraded in the cells (*μ*/*k*) within the total intracellular viral RNA produced (Fig 4A). Using all accepted MCMC parameter estimates from the time-course experimental datasets, we calculated that the fractions of viral RNA remaining were 31.6% and 25.0%, the fractions of viral RNA degraded were 67.7% and 73.3%, and the fractions of viral RNA released were 0.629% and 1.70% for JFH-1 and Jc1-n, respectively (Fig 4B). Comparing the lattermost fractions, Jc1-n used intracellular viral RNA 2.70 times more for virus release than JFH-1, explaining the rapid transmission of Jc1-n (S2B Fig). These results indicate the preferential “leaving-home” strategy of Jc1-n as compared with JFH-1, which adopts a “stay-at-home” strategy.

To further investigate these two opposing strategies, we addressed the relevance of viral RNA release rates for viral proliferation using in silico analysis. With various values of the release rate of intracellular viral RNA, *ρ*, we calculated the Malthusian parameter (Eq 7) for each strain as an indicator of viral fitness (Fig 4C). Each curve shows Malthusian parameters calculated using 100 parameter sets sampled from MCMC parameter estimates as functions of *ρ*, and each gray vertical line is the corresponding estimated release rate. Interestingly, the smaller the release rate, the larger the Malthusian parameter HCV achieves. This is because intracellular viral RNAs can be amplified faster compared with viral RNAs outside of cells that are degraded or enter new cells. This result showed that the JFH-1 strain is more optimized in terms of its Malthusian parameter compared with Jc1-n because of the smaller estimated values of *ρ*. That is, HCV JFH-1 adopts the stay-at-home strategy for acquiring a higher initial growth rate.

Next, we defined the cumulative number of newly infected cells at time *t* to evaluate viral transmissibility:

$$\int_{0}^{t}\beta T(\tau )V(\tau )d\tau =\int_{0}^{t}\beta_{\theta }T(\tau )V_{\theta }(\tau )d\tau .$$ (8)

We also calculated the cumulative number of newly infected cells for each strain using the means of the estimated parameters as functions of *ρ* (Fig 4D). Each curve shows the calculated cumulative numbers of infected cells until 3, 5, 7, 9, and 11 days post infection, and the gray vertical line represents the mean release rate estimated from the infection experiment. The value of the release rate, which maximized the cumulative number of newly infected cells, was between 0.1 and 0.5. This is because an intermediate release rate effectively increases extracellular viral RNA for new infection: lower release rates do not effectively produce new infections, whereas higher release rates decrease intracellular viral RNA levels and thus diminish future new infections. Thus, it appears that Jc1-n is more optimized for producing newly infected cells. This implies that HCV Jc1-n adopts the leaving-home strategy to acquire an advantage in producing newly infected cells.

Taken together, our theoretical investigation based on viral infection experiments revealed that the JFH-1 strain optimizes its initial growth rate, but the Jc1-n strain optimizes de novo infection. Ours is the first report to quantitatively evaluate these opposing evolutionary strategies and to show their significance for virus proliferation at the intracellular and intercellular levels.

## Discussion

To understand the replication mechanisms of HCV and to develop effective drugs for the treatment of HCV, many mathematical modeling studies have been conducted. A mathematical model describing the intracellular dynamics of HCV replication in detail provided a framework to investigate HCV replication dynamics using the well-established HCV replicon system [16]. Another study combined data obtained from the HCV replicon system with an expanded mathematical model and revealed that the replication compartment and unknown host factors played important roles in the maintenance and control of viral RNAs in cells [17]. Another expanded and modified model using data from an HCV cell culture system enabled predictions of the dynamics of HCV infection and viral protein mechanisms [18]. A multiscale model was applied to data from patients treated with several drugs, based on a basic mathematical model of viral dynamics with age structures of HCV replication; this work provided new insights into viral decay dynamics and the mechanisms of action of anti-HCV drugs [19,20]. Other multiscale models, combining the detailed intracellular replication dynamics and basic infection dynamics, also explained the dynamics of viral loads in vivo [21].

In this study, through a combined experimental-theoretical approach, we analyzed the dynamics of the HCV life cycle using two related HCV strains, JFH-1 and Jc1-n, employing different particle assembly/release strategies. We quantified the intra- and intercellular viral dynamics of these strains by applying an age-structured multiscale model to time-course experimental data from an HCV infection cell culture assay (Fig 2 and Table 1): as in [10,11], we transformed the multiscale model formulated by PDEs to an identical multiscale ODE model (i.e., Eqs 2–6), and we estimated parameters shared between the PDE and ODE models. It is technically challenging to obtain experimental measurements with age information, but thanks to the estimated values of these common parameters, we managed to reconstruct age information for intracellular viral RNA (S2 Fig). We also derived values for standard parameters as previously reported. For example, the numbers of viruses produced per cell per day in our multiscale model, which correspond to *ρA*(*t*)/*I*(*t*) in Eqs 2–6, were 10^2^–10^3^ and 10^3^–10^4^ for JFH-1 and Jc1-n, respectively. These values are relatively high compared with previously estimated values [1], potentially because of the high efficiency of viral replication in our Huh7.5.1 cell culture system. Note that values of *ρ* for JFH-1 and Jc1-n were both estimated as small compared with previous estimates [19], although we confirmed these estimated viral release rates using independent single-cycle infection experiments. This discrepancy is likely due to differences among experimental systems or mathematical models of intracellular viral replication dynamics. Interestingly, comparing the calculated Malthusian parameters (i.e., Eq 7) and the cumulative number of newly infected cells (i.e., Eq 8) between the two strains (Fig 3E and 3F), we found that the JFH-1 strain had a higher initial growth rate but that Jc1 produced more de novo infections.

Based on our results, we propose two opposing strategies for viral proliferation: the “stay-at-home strategy” and the “leaving-home strategy.” From an evolutionary perspective, JFH-1 adopts a stay-at-home strategy and preferentially uses viral genomic RNA for increasing intracellular replication. In contrast, adopting a leaving-home strategy, Jc1-n uses more viral genomic RNA for producing progeny virions capable of new transmission events to increase the number of infected cells (Fig 4). Thus, Jc1-n infects cells 1.82 times faster and produces viral RNA from infected cells 2.70 times faster than JFH-1. In the context of the replication-release trade-off, a previous theoretical study indicated that allocation and reallocation of intracellular viral RNA toward translation and replication rather than virus release induced higher viral production [22]. Although our model did not describe intracellular dynamics in detail, the “stay-at-home strategy” of JFH-1, resulting in more efficient accumulation of viruses by remaining in cells, was partially consistent with the results of previous work. Our group and others reported that JFH-1 assembled progeny virions on the membranes of hepatic lipid droplets, whereas J6/JFH-1 chimeric strains mainly used endoplasmic reticulum–derived membranes for particle production [2,3]. Although the molecular aspects of this difference have been analyzed, its significance for viral proliferation and dynamics is not completely understood. Our results raise the possibility that different subcellular locations for particle assembly and subsequent differential intracellular trafficking impact the rates of particle assembly and release, which in turn determine virus proliferation. Further analysis might shed light on why one HCV strain has to be assembled on the lipid droplet membrane whereas another assembles in association with the endoplasmic reticulum.

We confirmed that the replication rate and release rate can be approximated as constant regardless of infection age using independent single-cycle experiments. Thus, we assumed the kinetic parameters of intracellular replication were constant, which is a potential limitation of our study. To further validate this assumption, we confirmed that the numerical solution of the multiscale model with time-dependent intracellular kinetic parameters coincided well with that of our multiscale model (i.e., constant kinetic parameters). In addition, by comparison with previous models describing the HCV replicon, HCV cell culture systems, or in vivo data [16–18,21], we excluded the detailed biological processes of HCV replication to enable simple and robust parameter estimation. Thus, our tightly designed multicycle infection experiments and multiscale mathematical model enabled us to evaluate intracellular strategies of HCV strains on the basis of various aspects such as viral fitness and transmissibility, which together reflected the entire infection process.

The choice of replication strategy not only determines virus proliferation but also affects the pathogenic features of the virus: JFH-1, which preferentially amplifies intracellular RNA, caused fulminant hepatitis with rapid viral replication and severe inflammation. By contrast, J6, the original strain encoding the Jc1-n structural region, was isolated from a patient with chronic hepatitis and generally replicates more moderately, with robust spread of infected cells used as a longer-term strategy to establish persistent infection. Characterization of the proliferation strategies of viruses is of significant importance when trying to understand their clinical as well as evolutionary properties.

## Methods

### Cell culture and HCV infection

Huh-7.5.1 (kindly provided by Dr. Francis Chisari, The Scripps Research Institute) and Huh7-25 cells were cultured in Dulbecco’s Modified Eagle’s Medium (Invitrogen) supplemented with 10% fetal bovine serum (Sigma), 10 units/mL penicillin, 10 mg/mL streptomycin, 0.1 mM nonessential amino acids (Invitrogen), 1 mM sodium pyruvate, and 10 mM HEPES (pH 7.4) at 37°C under a humidified atmosphere containing 5% CO_2_. We used HCV strains JFH-1, a genotype 2a clinical isolate from a patient with fulminant hepatitis [4], and Jc1-n, a J6/JFH-1 chimeric laboratory strain [13]. JFH-1 and Jc1-n have 96.7% amino acid identity over the whole genome. HCV inoculum for infection experiments was recovered from the culture supernatants of Huh-7.5.1 cells transfected with the corresponding HCV RNA as described [4]. Huh-7.5.1 cells were inoculated with JFH-1 or Jc1-n at a multiplicity of infection (MOI) of 0.05 for 4 hours and then passaged to seed a new 96-well plate at different cell densities (1,000, 2,000, or 4,000 cells/well). Under these conditions, the starting number of HCV-infected cells varied. At days 0, 1, 2, 3, and 4 post seeding, culture supernatants and cell lysates were recovered to quantify HCV RNA by real-time RT-PCR as previously described [13]. The infectivity of HCV in culture supernatants was measured using a focus-forming assay as described [13]. To quantify the number of uninfected and infected cells, cells were fixed and stained with anti-HCV core antibody by immunofluorescence assay as described [13]. We prepared three replicate samples for these experiments and used the average values of replicates for analysis.

### Data fitting and parameter estimation

The parameters *g* and *K* were separately estimated (see S4 Text) and fixed at 0.670 per day and 4.12×10^4^ cells, respectively, for the JFH-1 strain and 0.665 per day and 3.75×10^4^ cells, respectively, for the Jc1-n strain. A statistical model adopted from Bayesian inference assumed that measurement error followed a normal distribution with mean zero and constant variance (error variance). The noninformative prior distributions with upper and lower limits were used as the prior distributions of parameters. The posterior predictive parameter distribution as an output of MCMC computation represented parameter variability. Distributions of model parameters (i.e., *β*_*θ*_, *k*, *ρ*, *f*_*θ*_) and initial values (i.e., *T*(0), *I*(0), *A*(0), *V*_*θ*_(*t*), *V*(0)) in Eqs 2–6 were inferred directly by MCMC computations. We also tested structural identifiability for all parameter estimates by calculating profile likelihood [23,24] using package dMod [25] in R Statistical Software [26]. All parameter estimates were confirmed to be structural identifiable (see S4 Fig). Distributions of derived quantities were calculated from the inferred parameter sets (Fig 3E and 3F for graphical representation). A set of computations for Eqs 2–6 with estimated parameter sets gives a distribution of outputs (the number of cells and the intra- and extracellular viral loads) as model predictions. To investigate variation in model predictions, global sensitivity analyses were performed. The ranges of possible variation are shown in Fig 2 as 95% posterior predictive intervals. Technical details of MCMC computations are summarized below.

### Statistical analysis

Package FME [27] in R Statistical Software [26] was used to infer posterior predictive parameter distributions. The delayed rejection and Metropolis method [28] was used as a default computation scheme for FME to perform MCMC computations. MCMC computations for parameter inference were implemented using the predefined function modMCMC() in package FME as introduced in **Methods**. Convergence of Markov chains to a stationary distribution was required to ensure parameter sets were sampled from a posterior distribution. Only the last 90,000 of 100,000 chains were used as burn-in. The convergence of the last 90,000 chains was manually checked with figures produced by package coda [29], a collection of diagnostic tools for MCMC computation. The 95% posterior predictive intervals shown as a shadowed region in each panel of Fig 2 were produced from 100 randomly chosen inferred parameter sets and corresponding model predictions. We employed a bootstrap *t* test [30] to quantitatively characterize differences in parameters and derived quantities between HCV JFH-1 and Jc1-n. In total, 100,000 parameter sets were sampled with replacement from the posterior predictive distributions to calculate the bootstrap t-statistics. To avoid potential sampling bias, the bootstrap *t* test was performed 100 times repeatedly. The averages of the computed *p*-values were used as indicators of differences.

## Supporting information

S1 Fig Parameter estimations from separate experiments. **(A)** Decay of extracellular HCV was estimated. HCV JFH-1 and Jc1-n were incubated in medium without cells and recovered at days 0, 1, 2, 3, 4, and 5 to quantify viral RNA and infectivity. Linear regressions yielded a rate of RNA degradation and a loss of virion infectivity per day. **(B)** Effect of changing medium on the clearance of extracellular viral RNA was estimated. Changing medium reduced viral RNA by 69.1% and 83.7% for JFH-1 and Jc1-n, respectively, and these losses were modeled by approximating the sampling of virus as a continuous exponential decay (S1 Protocol and S5 Text). **(C)** Decay kinetics of intracellular viral RNA was investigated upon complete inhibition of RNA replication/release by antiviral treatment with 2 μM SOF + 1 μM LDV. By applying linear regressions, the degradation rates of intracellular viral RNA for JFH-1 and Jc1-n were estimated. **(D)** By counting total Huh7.5.1 cells on days 0, 1, 2, 3, 4 in experiments A, B, and C, the growth kinetics of the cells were estimated. The underlying data for this figure can be found in S4 Data. HCV, hepatitis C virus; LDV, ledipasvir; SOF, sofosbuvir.
(DOCX)

S2 Fig Parameter estimations from separate experiments. **(A)** Dynamics of the distributions of intracellular viral RNA according to infection age, *a*. The distributions were calculated using the original multiscale PDE model (Eq S2–6 in S1 Text) using the means of parameter estimates for HCV JFH-1 and Jc1-n. The colored bars represent amounts of intracellular viral RNA. **(B)** Differences in the distribution of intracellular viral RNA in total infected cells of infection age, *a*, between HCV JFH-1 and Jc1-n. The colored bar shows the difference in the amount of intracellular viral RNA (green: intracellular viral RNA during Jc1-n infection is more abundant than during JFH-1 infection; yellow-red-brown: intracellular viral RNA is more abundant for JFH-1 than for Jc1-n; gray: no new infection occurs owing to depletion of target cells). The underlying data for this figure can be found in S5 Data. HCV, hepatitis C virus; PDE, partial differential equation.
(DOCX)

S3 Fig Validation of differences in viral entry and release between JFH-1 and Jc1-n. **(A)** Difference in viral entry mediated by the envelopes of JFH-1 and J6 (structural region of Jc1-n). HCVtcp prepared with an HCV E1/E2 derived from JFH-1 and J6 were used to inoculate Huh7.5.1 cells. At 72 hours post inoculation, luciferase activity was measured to evaluate differences in viral entry between JFH-1 and Jc1-n according to previous work [31]. **(B)** A single-cycle virus production assay was performed by transfecting Huh7-25 cells with JFH-1 or Jc1-n RNA. Huh7-25 cells are deficient for an HCV receptor, CD81, and do not support reinfection [32]. HCV RNA produced in the culture supernatant at 72 hours post transfection was quantified by real-time RT-PCR to evaluate viral production. **(C)** Single-cycle virus production assay for examining early HCV replication. Production of HCV core and NS5A proteins acted as an internal control and was detected by immunoblotting of Huh7-25 cells transfected with RNA derived from either JFH-1 or Jc1-n at early time points (16, 20, 25, and 30 hours post transfection). **(D)** Host IFN response against infection by JFH-1 and Jc1-n. Huh7.5.1 cells were infected with either JFH-1 or Jc1-n, and induction of IFN-stimulated genes (MxA, and ISG56) and expression of HCV core, NS5A, and actin proteins were assessed by immunoblotting. Exogenous IFN-α was used as a positive control for ISG induction. **(E)** Expression of host factors regulating HCV particle assembly switching in JFH-1- and Jc1-n-infected cells at different infection ages. YTHDF1, 2, 3, and METTL14 as well as actin were detected in JFH-1- and Jc1-n-infected cells at infection ages of 3, 5, and 7 days by immunoblotting. The underlying data for this figure can be found in S6 Data. E, envelope; HCV, hepatitis C virus; HCVtcp, trans-complemented HCV particles; IFN, interferon; ISG, interferon stimulated genes; METTL14, methyltransferase Like 14; RT-PCR, reverse transcription PCR.
(DOCX)

S4 Fig Profile likelihood of estimated parameters for identifiability analysis. All estimated parameters were confirmed to be structurally identifiable by calculation of profile likelihood [23–25].
(DOCX)

S1 Table Fitted initial (t = 0) values for the in vitro experiment. (DOCX)

S1 Data Original numerical values for Fig 2. (XLSX)

S2 Data Original numerical values for Fig 3. (XLSX)

S3 Data Original numerical values for Fig 4. (XLSX)

S4 Data Original numerical values for S1 Fig. (XLSX)

S5 Data Original numerical values for S2 Fig. (XLSX)

S6 Data Original numerical values for S3 Fig. (XLSX)

S1 Raw Images Original raw images for S3C, S3D and S3E Fig. (ZIP)

S1 Protocol Wash assay. (DOCX)

S2 Protocol Single-round entry assay. (DOCX)

S3 Protocol Single-cycle HCV production assay. HCV, hepatitis C virus.
(DOCX)

S1 Text Transformation to a system of ODEs from a PDE multiscale model. ODE, ordinary differential equation; PDE, partial differential equation.
(DOCX)

S2 Text Validation of the difference in virus entry and production between JFH-1 and Jc1-n. (DOCX)

S3 Text Derivation of the Malthusian parameter. (DOCX)

S4 Text Quantitation of cell growth. (DOCX)

S5 Text Quantitation of clearance rate due to washing. (DOCX)

## Abbreviations

ffu: focus formation unit
HCV: hepatitis C virus
MCMC: Markov chain Monte Carlo
NS protein: nonstructural protein
ODE: ordinary differential equation
PDE: partial differential equation
S protein: structural protein
