# Supplementary material for: Should a viral genome stay in the host cell or leave? A quantitative dynamics study of how hepatitis C virus deals with this dilemma
Source: PLoS Biol. 2020 Jul 30;18(7):e3000562. doi: 10.1371/journal.pbio.3000562 (PMC7392214; doi:10.1371/journal.pbio.3000562)
Supplement: S1 Fig — (A) Decay of extracellular HCV was estimated. HCV JFH-1 and Jc1-n were incubated in medium without cells and recovered at days 0, 1, 2, 3, 4, and 5 to quantify viral RNA and infectivity. Linear regressions yielded a rate of RNA degradation and a loss of virion infectivity per day. (B) Effect of changing medium on the clearance of extracellular viral RNA was estimated. Changing medium reduced viral RNA by 69.1% and 83.7% for JFH-1 and Jc1-n, respectively, and these losses were modeled by approximating the sampling of virus as a continuous exponential decay (S1 Protocol and S5 Text). (C) Decay kinetics of intracellular viral RNA was investigated upon complete inhibition of RNA replication/release by antiviral treatment with 2 μM SOF + 1 μM LDV. By applying linear regressions, the degradation rates of intracellular viral RNA for JFH-1 and Jc1-n were estimated. (D) By counting total Huh7.5.1 cells on days 0, 1, 2, 3, 4 in experiments A, B, and C, the growth kinetics of the cells were estimated. The underlying data for this figure can be found in S4 Data. HCV, hepatitis C virus; LDV, ledipasvir; SOF, sofosbuvir. (DOCX) [file pbio.3000562.s001.docx]

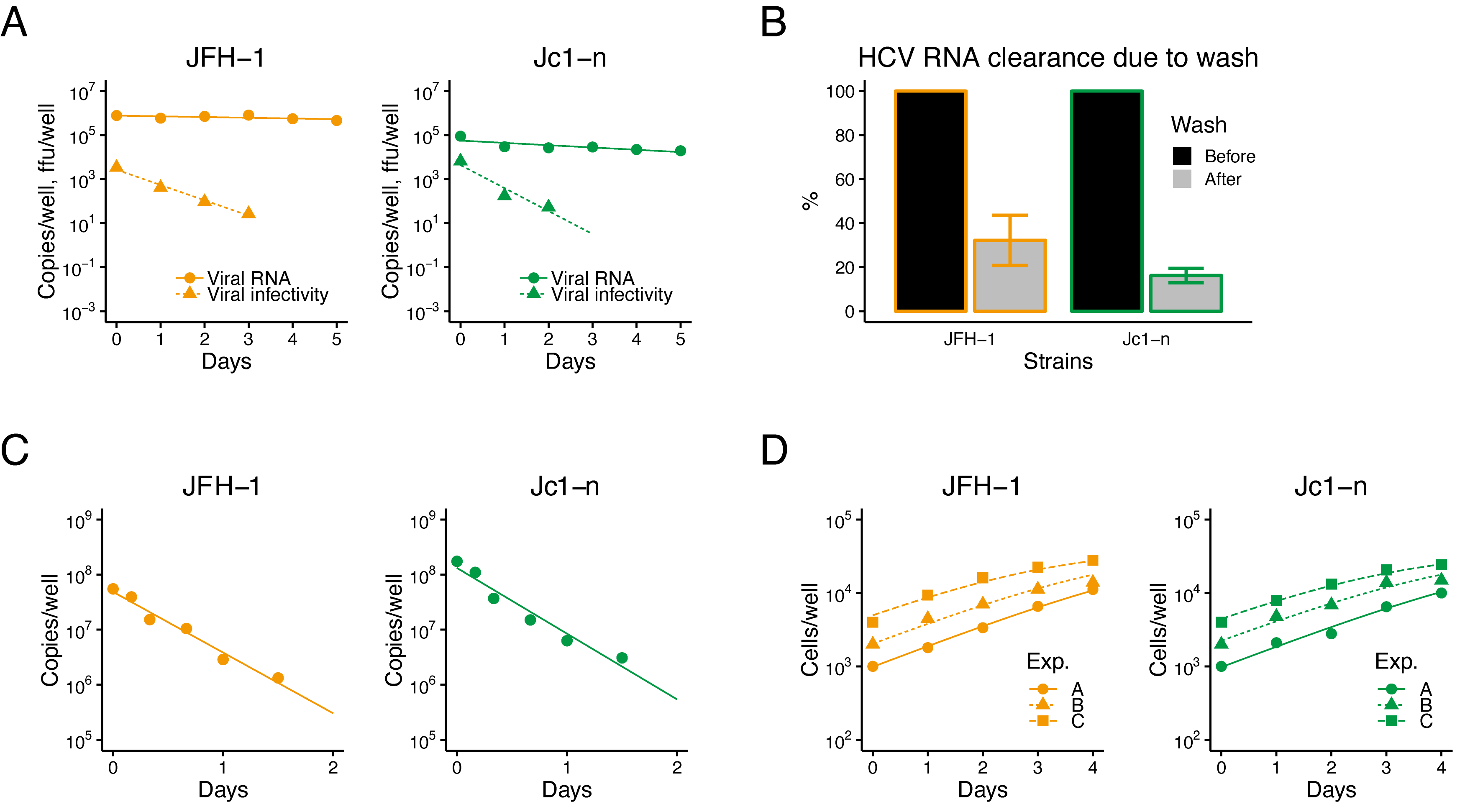


**S1 Fig.** **Parameter estimations from separate experiments: (A)** Decay of extracellular HCV was estimated. HCV JFH-1 and Jc1-n were incubated in medium without cells and recovered at days 0, 1, 2, 3, 4, and 5 to quantify viral RNA and infectivity. Linear regressions yielded a rate of RNA degradation and a loss of virion infectivity per day. **(B)** Effect of changing medium on the clearance of extracellular viral RNA was estimated. Changing medium reduced viral RNA by 69.1% and 83.7% for JFH-1 and Jc1-n, respectively, and these losses were modeled by approximating the sampling of virus as a continuous exponential decay (**S1 Protocol** and **S5 Text**). **(C)** Decay kinetics of intracellular viral RNA was investigated upon complete inhibition of RNA replication/release by antiviral treatment with 2 µM sofosbuvir (SOF) + 1 µM ledipasvir (LDV). By applying linear regressions, the degradation rates of intracellular viral RNA for JFH-1 and Jc1-n were estimated. **(D)** By counting total Huh7.5.1 cells on days 0, 1, 2, 3, 4 in Experiments A, B and C, the growth kinetics of the cells were estimated. The underlying data for this Figure can be found in S4_Data.
