# Supplementary material for: Should a viral genome stay in the host cell or leave? A quantitative dynamics study of how hepatitis C virus deals with this dilemma
Source: PLoS Biol. 2020 Jul 30;18(7):e3000562. doi: 10.1371/journal.pbio.3000562 (PMC7392214; doi:10.1371/journal.pbio.3000562)
Supplement: S2 Fig — (A) Dynamics of the distributions of intracellular viral RNA according to infection age, a. The distributions were calculated using the original multiscale PDE model (Eq S2–6 in S1 Text) using the means of parameter estimates for HCV JFH-1 and Jc1-n. The colored bars represent amounts of intracellular viral RNA. (B) Differences in the distribution of intracellular viral RNA in total infected cells of infection age, a, between HCV JFH-1 and Jc1-n. The colored bar shows the difference in the amount of intracellular viral RNA (green: intracellular viral RNA during Jc1-n infection is more abundant than during JFH-1 infection; yellow-red-brown: intracellular viral RNA is more abundant for JFH-1 than for Jc1-n; gray: no new infection occurs owing to depletion of target cells). The underlying data for this figure can be found in S5 Data. HCV, hepatitis C virus; PDE, partial differential equation. (DOCX) [file pbio.3000562.s002.docx]

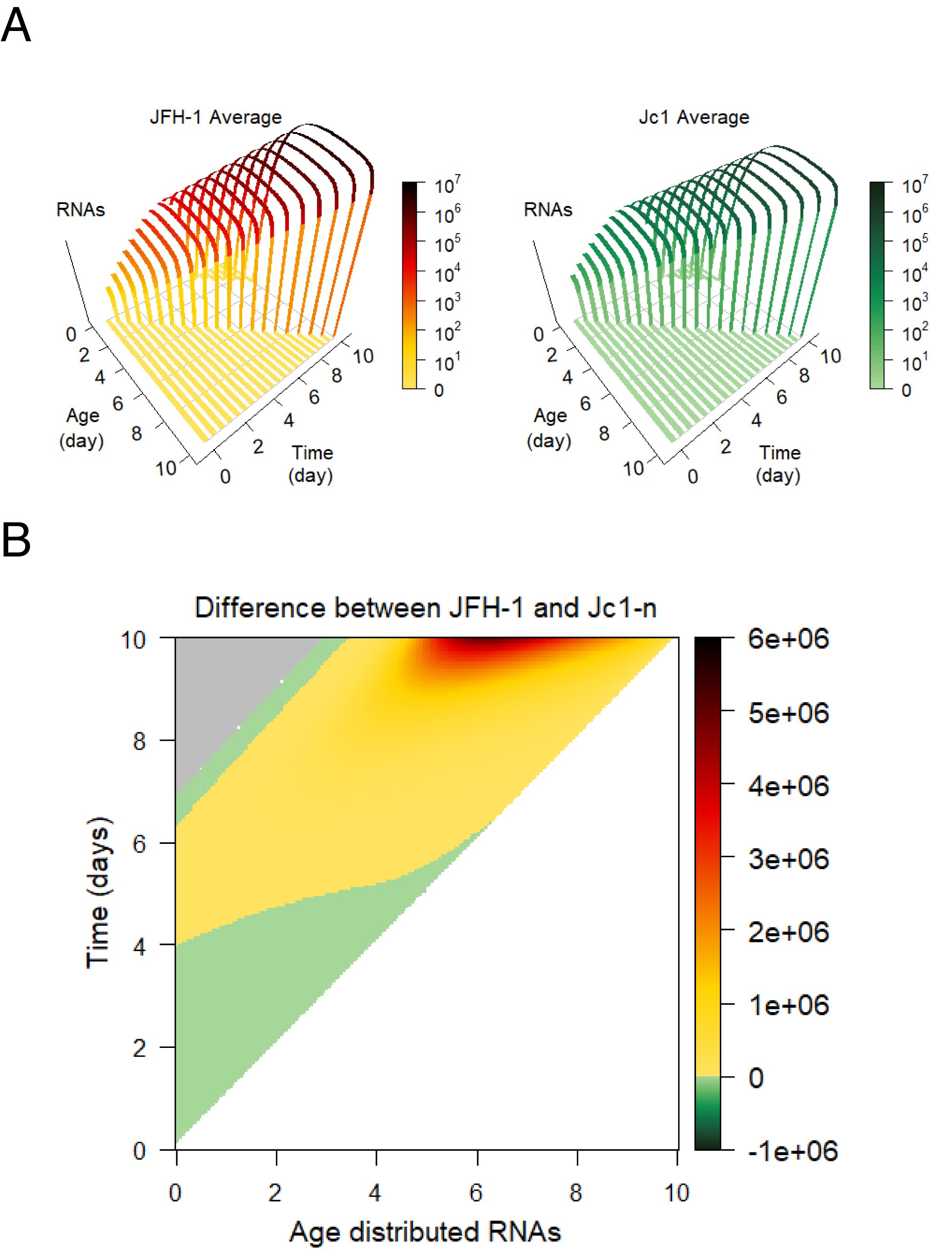


**S2 Fig.** **Parameter estimations from separate experiments: (A)** Dynamics of the distributions of intracellular viral RNA according to infection age, $a$. The distributions were calculated using the original multiscale PDE model (Eqs. (S2–6) in **S1 Text**) using the means of parameter estimates for HCV JFH-1 and Jc1-n. The colored bars represent amounts of intracellular viral RNA. **(B)** Differences in the distribution of intracellular viral RNA in total infected cells of infection age, $a$, between HCV JFH-1 and Jc1-n. The colored bar shows the difference in the amount of intracellular viral RNA (green: intracellular viral RNA during Jc1-n infection is more abundant than during JFH-1 infection; yellow-red-brown: intracellular viral RNA is more abundant for JFH-1 than for Jc1-n; gray: no new infection occurs owing to depletion of target cells). The underlying data for this Figure can be found in S5_Data.
