# Supplementary material for: Should a viral genome stay in the host cell or leave? A quantitative dynamics study of how hepatitis C virus deals with this dilemma
Source: PLoS Biol. 2020 Jul 30;18(7):e3000562. doi: 10.1371/journal.pbio.3000562 (PMC7392214; doi:10.1371/journal.pbio.3000562)
Supplement: S3 Fig — (A) Difference in viral entry mediated by the envelopes of JFH-1 and J6 (structural region of Jc1-n). HCVtcp prepared with an HCV E1/E2 derived from JFH-1 and J6 were used to inoculate Huh7.5.1 cells. At 72 hours post inoculation, luciferase activity was measured to evaluate differences in viral entry between JFH-1 and Jc1-n according to previous work [31]. (B) A single-cycle virus production assay was performed by transfecting Huh7-25 cells with JFH-1 or Jc1-n RNA. Huh7-25 cells are deficient for an HCV receptor, CD81, and do not support reinfection [32]. HCV RNA produced in the culture supernatant at 72 hours post transfection was quantified by real-time RT-PCR to evaluate viral production. (C) Single-cycle virus production assay for examining early HCV replication. Production of HCV core and NS5A proteins acted as an internal control and was detected by immunoblotting of Huh7-25 cells transfected with RNA derived from either JFH-1 or Jc1-n at early time points (16, 20, 25, and 30 hours post transfection). (D) Host IFN response against infection by JFH-1 and Jc1-n. Huh7.5.1 cells were infected with either JFH-1 or Jc1-n, and induction of IFN-stimulated genes (MxA, and ISG56) and expression of HCV core, NS5A, and actin proteins were assessed by immunoblotting. Exogenous IFN-α was used as a positive control for ISG induction. (E) Expression of host factors regulating HCV particle assembly switching in JFH-1- and Jc1-n-infected cells at different infection ages. YTHDF1, 2, 3, and METTL14 as well as actin were detected in JFH-1- and Jc1-n-infected cells at infection ages of 3, 5, and 7 days by immunoblotting. The underlying data for this figure can be found in S6 Data. E, envelope; HCV, hepatitis C virus; HCVtcp, trans-complemented HCV particles; IFN, interferon; ISG, interferon stimulated genes; METTL14, methyltransferase Like 14; RT-PCR, reverse transcription PCR. (DOCX) [file pbio.3000562.s003.docx]

**S3 Fig.** **Validation of differences in viral entry and release between JFH-1 and Jc1-n: (A)** Difference in viral entry mediated by the envelopes of JFH-1 and J6 (structural region of Jc1-n). Trans-complemented HCV particles (HCVtcp) prepared with an HCV envelope (E)1/E2 derived from JFH-1 and J6 were used to inoculate Huh7.5.1 cells. At 72 h post-inoculation, luciferase activity was measured to evaluate differences in viral entry between JFH-1 and Jc1-n according to previous work [1]. **(B)** A single-cycle virus production assay was performed by transfecting Huh7-25 cells with JFH-1 or Jc1-n RNA. Huh7-25 cells are deficient for an HCV receptor, CD81, and do not support re-infection [2]. HCV RNA produced in the culture supernatant at 72 h post-transfection was quantified by real time RT-PCR to evaluate viral production. **(C)** Single-cycle virus production assay for examining early HCV replication. Production of HCV core and NS5A proteins acted as an internal control and was detected by immunoblotting of Huh7-25 cells transfected with RNA derived from either JFH-1 or Jc1-n at early time points (16, 20, 25 and 30 h post-transfection). **(D)** Host interferon (IFN) response against infection by JFH-1 and Jc1-n. Huh7.5.1 cells were infected with either JFH-1 or Jc1-n, and induction of IFN-stimulated genes (MxA, and ISG56) and expression of HCV core, NS5A, and actin proteins were assessed by immunoblotting. Exogenous IFN-α was used as a positive control for ISG induction. **(E)** Expression of host factors regulating HCV particle assembly switching in JFH-1- and Jc1-n-infected cells at different infection ages. YTHDF1, 2, 3, and METTL14 as well as actin were detected in JFH-1- and Jc1-n-infected cells at infection ages of 3, 5, and 7 days by immunoblotting. The underlying data for this Figure can be found in S6_Data and the original raw images for S3C-S4E Fig can be found in S1_Raw_Images.

**Supplementary References**

1. Suzuki R, Saito K, Kato T, Shirakura M, Akazawa D, Ishii K, et al. Trans-complemented hepatitis C virus particles as a versatile tool for study of virus assembly and infection. Virology. 2012;432(1):29-38. Epub 2012/06/26. doi: 10.1016/j.virol.2012.05.033. PubMed PMID: 22727832.

2. Kato T, Choi Y, Elmowalid G, Sapp RK, Barth H, Furusaka A, et al. Hepatitis C virus JFH-1 strain infection in chimpanzees is associated with low pathogenicity and emergence of an adaptive mutation. Hepatology. 2008;48(3):732-40. Epub 2008/08/21. doi: 10.1002/hep.22422. PubMed PMID: 18712792; PubMed Central PMCID: PMCPMC2535917.
