# Supplementary material for: Should a viral genome stay in the host cell or leave? A quantitative dynamics study of how hepatitis C virus deals with this dilemma
Source: PLoS Biol. 2020 Jul 30;18(7):e3000562. doi: 10.1371/journal.pbio.3000562 (PMC7392214; doi:10.1371/journal.pbio.3000562)
Supplement: S4 Fig — All estimated parameters were confirmed to be structurally identifiable by calculation of profile likelihood [23–25]. (DOCX) [file pbio.3000562.s004.docx]

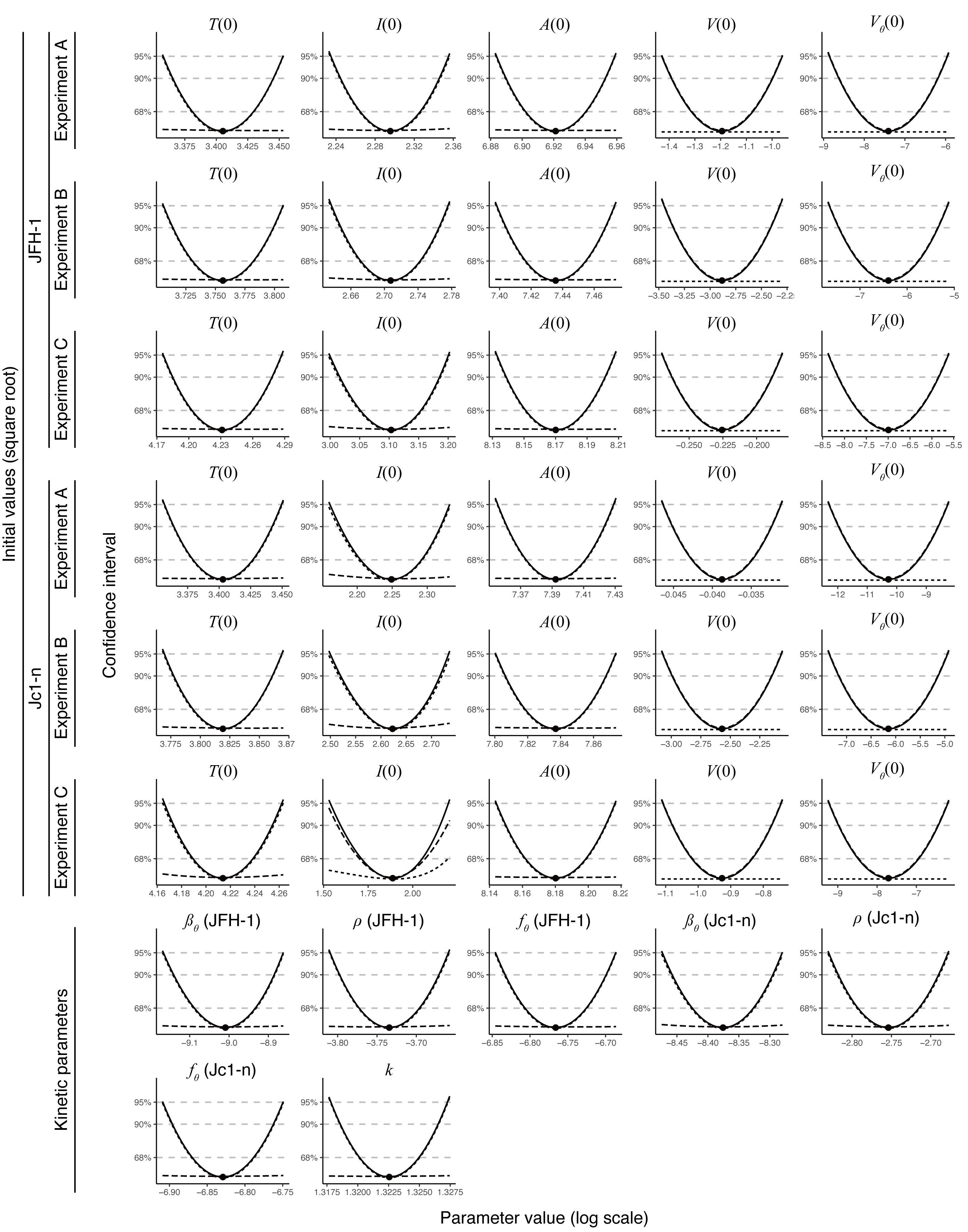


**S4 Fig.** **Profile likelihood of estimated parameters for identifiability analysis:** All estimated parameters were confirmed to be structurally identifiable by calculation of profile likelihood [1-3].

**Supplementary References**

1. Raue A, Kreutz C, Maiwald T, Bachmann J, Schilling M, Klingmuller U, et al. Structural and practical identifiability analysis of partially observed dynamical models by exploiting the profile likelihood. Bioinformatics. 2009;25(15):1923-9. Epub 2009/06/10. doi: 10.1093/bioinformatics/btp358. PubMed PMID: 19505944.

2. Maiwald T, Hass H, Steiert B, Vanlier J, Engesser R, Raue A, et al. Driving the Model to Its Limit: Profile Likelihood Based Model Reduction. PLoS One. 2016;11(9):e0162366. Epub 2016/09/03. doi: 10.1371/journal.pone.0162366. PubMed PMID: 27588423; PubMed Central PMCID: PMCPMC5010240 support in the form of salaries from Merrimack Pharmaceuticals. This does not alter the authors' adherence to PLOS ONE policies on sharing data and materials.

3. Kaschek D, Mader W, Fehling-Kaschek M, Rosenblatt M, Timmer J. Dynamic Modeling, Parameter Estimation, and Uncertainty Analysis in R. Journal of Statistical Software. 2019;88(10):1-32. doi: 10.18637/jss.v088.i10. PubMed PMID: WOS:000467010500001.
