# Supplementary material for: Should a viral genome stay in the host cell or leave? A quantitative dynamics study of how hepatitis C virus deals with this dilemma
Source: PLoS Biol. 2020 Jul 30;18(7):e3000562. doi: 10.1371/journal.pbio.3000562 (PMC7392214; doi:10.1371/journal.pbio.3000562)
Supplement: S1 Table — (DOCX) [file pbio.3000562.s005.docx]

**S1 Table |** Fitted initial (t=0) values for the *in vitro* experiment.

| Variable | Unit | Fitted initial value at the indicated number of plated cells (95% confidence interval) | | | | | |
| --- | --- | --- | --- | --- | --- | --- | --- |
|  |  | HCV JFH-1 | | | HCV Jc1-n | | |
|  |  | 1000  (plated cells/well) | 2000  (plated cells/well) | 4000  (plated cells/well) | 1000  (plated cells/well) | 2000  (plated cells/well) | 4000  (plated cells/well) |
| $T\left( 0 \right)$ | cells/well | $9.90\times{10}^{2}$  ($\left( 5.72-1.64 \right)\times{10}^{3}$) | $1.69\times{10}^{3}$  ($\left( 0.992-3.49 \right)\times{10}^{3}$) | $5.14\times{10}^{3}$  ($\left( 2.54-9.56 \right)\times{10}^{3}$) | $9.19\times{10}^{2}$  ($\left( 0.595-1.35 \right)\times{10}^{3}$) | $2.27\times{10}^{3}$  ($\left( 1.00-4.57 \right)\times{10}^{3}$) | $4.57\times{10}^{3}$  ($\left( 2.87-6.93 \right)\times{10}^{3}$) |
| $I\left( 0 \right)$ | cells/well | $1.01\times{10}^{2}$  ($\left( 0.423-2.00 \right)\times{10}^{2}$) | $2.33\times{10}^{2}$  ($\left( 1.38-3.73 \right)\times{10}^{2}$) | $5.13\times{10}^{2}$  ($\left( 3.03-8.06 \right)\times{10}^{2}$) | $9.64\times10$  ($\left( 0.425-1.89 \right)\times{10}^{2}$) | $1.89\times{10}^{2}$  ($\left( 0.983-3.24 \right)\times{10}^{2}$) | $4.54\times10$  ($\left( 1.75-9.27 \right)\times10$) |
| $A\left( 0 \right)$ | RNA copies/well | $1.05\times{10}^{6}$  ($\left( 0.834-1.30 \right)\times{10}^{6}$) | $2.94\times{10}^{6}$  ($\left( 1.77-4.59 \right)\times{10}^{6}$) | $1.27\times{10}^{7}$  ($\left( 0.822-1.90 \right)\times{10}^{7}$) | $2.71\times{10}^{6}$  ($\left( 1.63-4.22 \right)\times{10}^{6}$) | $6.76\times{10}^{6}$  ($\left( 0.443-1.02 \right)\times{10}^{7}$) | $1.33\times{10}^{7}$  ($\left( 0.916-1.86 \right)\times{10}^{7}$) |
| $V\left( 0 \right)$ | RNA copies/well | $9.33\times{10}^{-2}$  ($\left( 5.44-1.52 \right)\times{10}^{-2}$) | $3.20\times{10}^{-3}$  ($\left( 1.64-5.59 \right)\times{10}^{-3}$) | $6.72\times{10}^{-1}$  ($0.241-1.50$) | $1.03$  ($0.230-2.92$) | $6.18\times{10}^{-3}$  ($\left( 0.222-1.37 \right)\times{10}^{-2}$) | $1.61\times{10}^{-1}$  ($\left( 0.833-2.84 \right)\times{10}^{-1}$) |
| $V_{\theta}\left( 0 \right)$ | ffu/well | $3.84\times{10}^{-7}$  ($\left( 1.66-7.74 \right)\times{10}^{-7}$) | $2.91\times{10}^{-6}$  ($\left( 1.19-6.19 \right)\times{10}^{-6}$) | $8.68\times{10}^{-7}$  ($\left( 0.401-1.68 \right)\times{10}^{-6}$) | $1.24\times{10}^{-9}$  ($\left( 0.421-2.83 \right)\times{10}^{-9}$) | $4.91\times{10}^{-6}$  ($\left( 0.148-1.21 \right)\times{10}^{-5}$) | $2.05\times{10}^{-7}$  ($\left( 1.08-3.49 \right)\times{10}^{-7}$) |
