# Supplementary figures and images for: Should a viral genome stay in the host cell or leave? A quantitative dynamics study of how hepatitis C virus deals with this dilemma
Source: PLoS Biol. 2020 Jul 30;18(7):e3000562. doi: 10.1371/journal.pbio.3000562 (PMC7392214; doi:10.1371/journal.pbio.3000562)

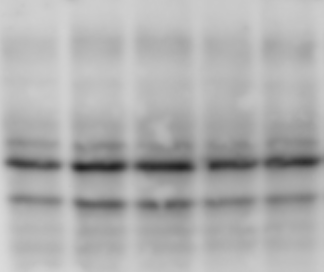

Supplement: S1 Raw Images — (ZIP) [file pbio.3000562.s012.zip › blot/Fig. S3C-actin Jc1-n.TIF]

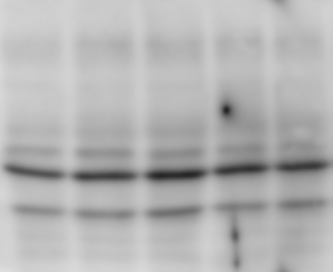

Supplement: S1 Raw Images — (ZIP) [file pbio.3000562.s012.zip › blot/Fig. S3C-actin JFH-1.TIF]

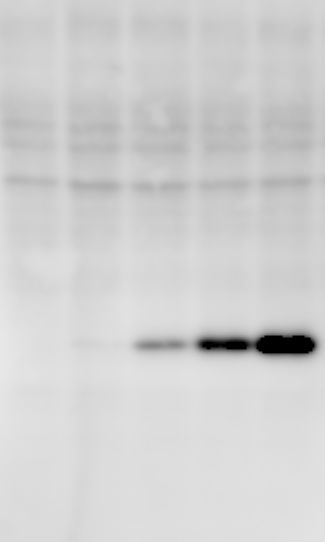

Supplement: S1 Raw Images — (ZIP) [file pbio.3000562.s012.zip › blot/Fig. S3C-HCV core Jc1-n.TIF]

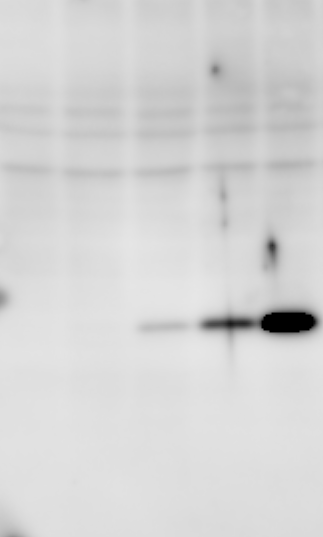

Supplement: S1 Raw Images — (ZIP) [file pbio.3000562.s012.zip › blot/Fig. S3C-HCV core JFH-1.TIF]

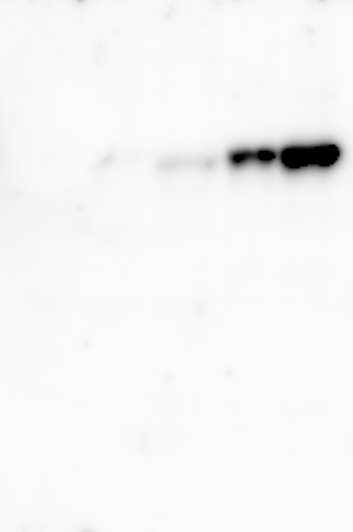

Supplement: S1 Raw Images — (ZIP) [file pbio.3000562.s012.zip › blot/Fig. S3C-HCV NS5A Jc1-n.TIF]

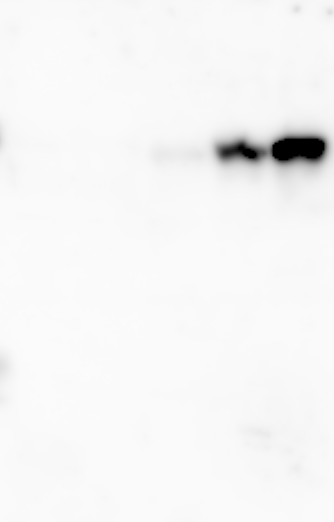

Supplement: S1 Raw Images — (ZIP) [file pbio.3000562.s012.zip › blot/Fig. S3C-HCV NS5A JFH-1.TIF]

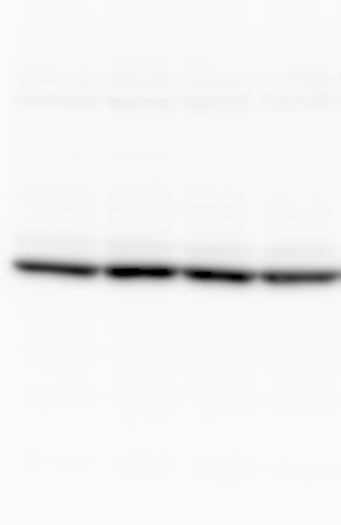

Supplement: S1 Raw Images — (ZIP) [file pbio.3000562.s012.zip › blot/Fig. S3D-actin.TIF]

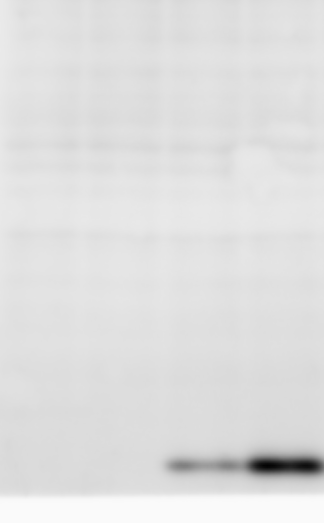

Supplement: S1 Raw Images — (ZIP) [file pbio.3000562.s012.zip › blot/Fig. S3D-HCVcore.TIF]

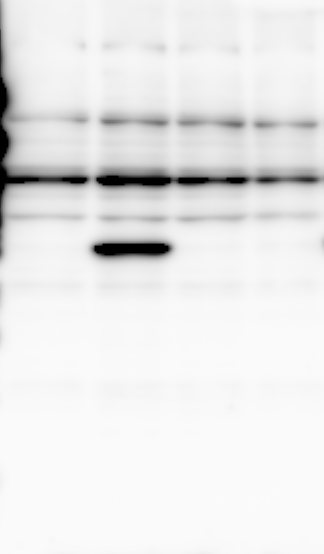

Supplement: S1 Raw Images — (ZIP) [file pbio.3000562.s012.zip › blot/Fig. S3D-ISG56.TIF]

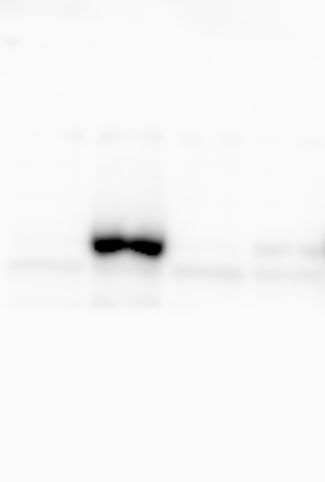

Supplement: S1 Raw Images — (ZIP) [file pbio.3000562.s012.zip › blot/Fig. S3D-MxA.TIF]

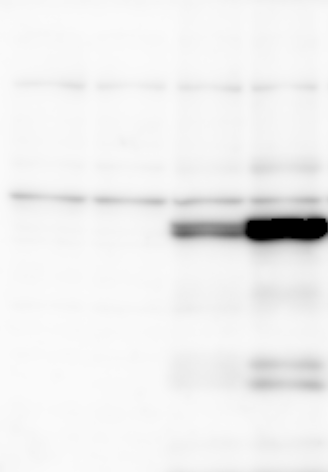

Supplement: S1 Raw Images — (ZIP) [file pbio.3000562.s012.zip › blot/Fig. S3D-NS5A.TIF]

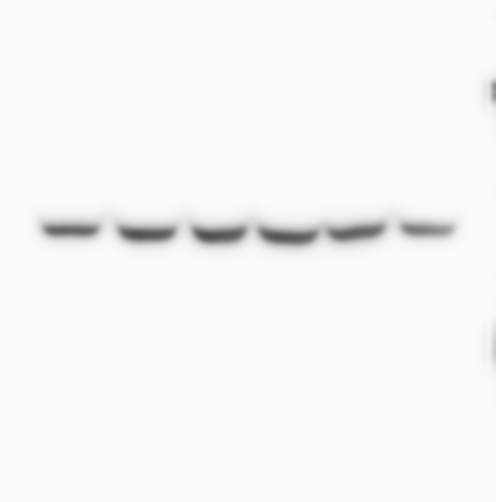

Supplement: S1 Raw Images — (ZIP) [file pbio.3000562.s012.zip › blot/Fig. S3E-actin.TIF]

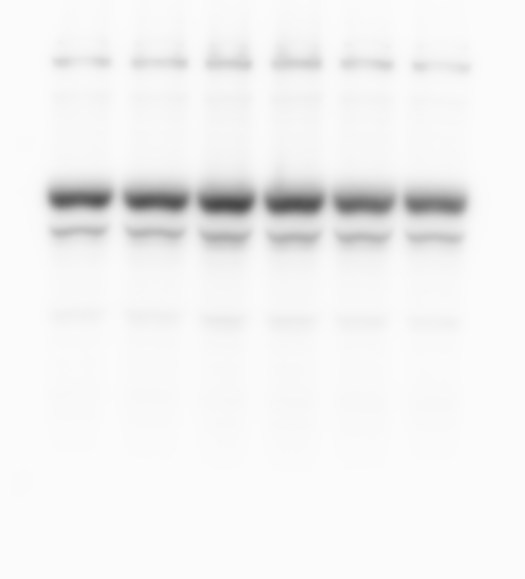

Supplement: S1 Raw Images — (ZIP) [file pbio.3000562.s012.zip › blot/Fig. S3E-METTL14.TIF]

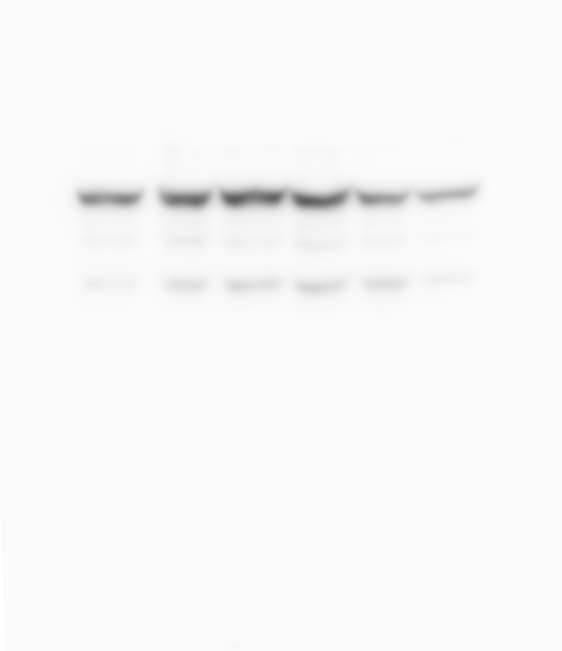

Supplement: S1 Raw Images — (ZIP) [file pbio.3000562.s012.zip › blot/Fig. S3E-YTHDF1.TIF]

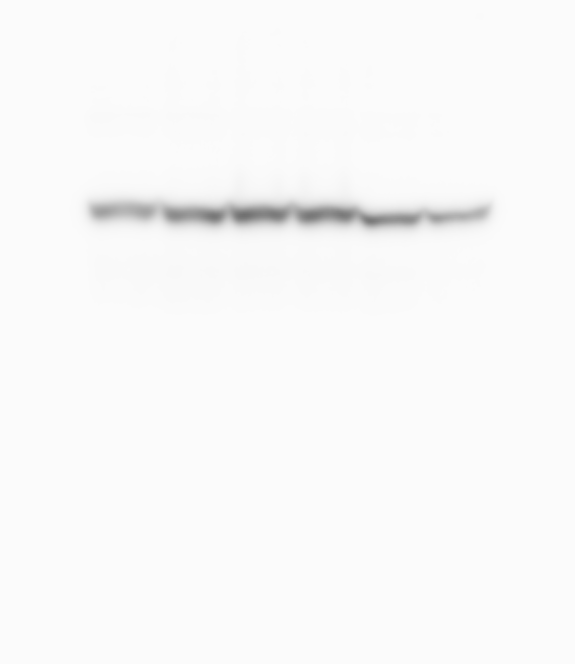

Supplement: S1 Raw Images — (ZIP) [file pbio.3000562.s012.zip › blot/Fig. S3E-YTHDF2.TIF]

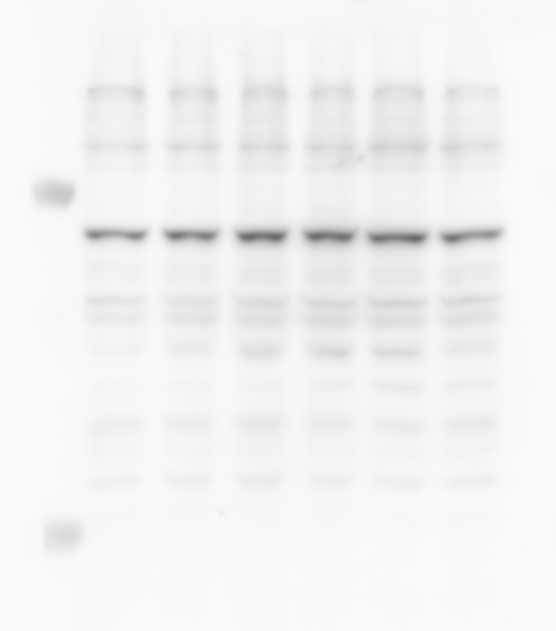

Supplement: S1 Raw Images — (ZIP) [file pbio.3000562.s012.zip › blot/Fig. S3E-YTHDF3.TIF]
