# Supplementary material for: Should a viral genome stay in the host cell or leave? A quantitative dynamics study of how hepatitis C virus deals with this dilemma
Source: PLoS Biol. 2020 Jul 30;18(7):e3000562. doi: 10.1371/journal.pbio.3000562 (PMC7392214; doi:10.1371/journal.pbio.3000562)
Supplement: S1 Protocol — (DOCX) [file pbio.3000562.s013.docx]

**S1 Protocol: Wash assay**

To examine HCV RNA clearance by medium washing, we removed virus-containing culture supernatants of Huh7.5.1 cells infected with either JFH-1 or Jc1-n and added fresh medium. HCV RNA was quantitated in the recovered culture supernatant by real time RT-PCR (before wash). The replenished new medium was also immediately recovered to quantify HCV RNA (after wash). The ratio of HCV RNA in the replenished medium to that in the culture supernatant before medium replenishment was calculated and shown as the HCV RNA clearance rate for JFH-1 and Jc1-n in Panel B, **S1** **Fig.**
