# Supplementary material for: Should a viral genome stay in the host cell or leave? A quantitative dynamics study of how hepatitis C virus deals with this dilemma
Source: PLoS Biol. 2020 Jul 30;18(7):e3000562. doi: 10.1371/journal.pbio.3000562 (PMC7392214; doi:10.1371/journal.pbio.3000562)
Supplement: S2 Protocol — (DOCX) [file pbio.3000562.s014.docx]

**S2 Protocol: Single-round entry assay**

In **S3A** **Fig**, trans-complemented HCV particles (HCVtcp) [1] were used for evaluating the HCV envelope-mediated entry process. HCVtcp was recovered from the culture supernatants of Huh7.5.1 cells transfected with expression vectors encoding HCV core to NS2 proteins and HCV subgenomic replicon RNA encoding the Gaussia luciferase gene upstream of the HCV non-structural region (SGR-Gluc/NS3m) [2]. Huh7.5.1 cells were inoculated with HCVtcp for 4 h, followed by washing out and incubating for an additional 72 h in fresh culture medium as described [1]. Luciferase activity in the supernatant was measured to assess HCV entry.

**Supplementary References**

1. Suzuki R, Saito K, Kato T, Shirakura M, Akazawa D, Ishii K, et al. Trans-complemented hepatitis C virus particles as a versatile tool for study of virus assembly and infection. Virology. 2012;432(1):29-38. Epub 2012/06/26. doi: 10.1016/j.virol.2012.05.033. PubMed PMID: 22727832.

2. Saga R, Fujimoto A, Watanabe N, Matsuda M, Hasegawa M, Watashi K, et al. Bivalent vaccine platform based on Japanese encephalitis virus (JEV) elicits neutralizing antibodies against JEV and hepatitis C virus. Sci Rep. 2016;6:28688. Epub 2016/06/28. doi: 10.1038/srep28688. PubMed PMID: 27345289; PubMed Central PMCID: PMCPMC4922013.
