# Supplementary material for: Should a viral genome stay in the host cell or leave? A quantitative dynamics study of how hepatitis C virus deals with this dilemma
Source: PLoS Biol. 2020 Jul 30;18(7):e3000562. doi: 10.1371/journal.pbio.3000562 (PMC7392214; doi:10.1371/journal.pbio.3000562)
Supplement: S3 Protocol — HCV, hepatitis C virus. (DOCX) [file pbio.3000562.s015.docx]

**S3 Protocol: Single-cycle HCV production assay**

In **S3B and S3C** **Fig**, single-cycle virus production assays were used to reproduce the replication to release steps in the HCV life cycle [1]. Huh7-25 cells were transfected with HCV JFH-1 or Jc1-n RNA. These cells lack an HCV entry receptor, CD81, and do not allow virus re-infection. At 72 h post-transfection, production of HCV in culture supernatants was evaluated by quantification of extracellular HCV RNA by real time RT-PCR as described [2].

**Supplementary References**

1. Kato T, Choi Y, Elmowalid G, Sapp RK, Barth H, Furusaka A, et al. Hepatitis C virus JFH-1 strain infection in chimpanzees is associated with low pathogenicity and emergence of an adaptive mutation. Hepatology. 2008;48(3):732-40. Epub 2008/08/21. doi: 10.1002/hep.22422. PubMed PMID: 18712792; PubMed Central PMCID: PMCPMC2535917.

2. Watashi K, Hijikata M, Hosaka M, Yamaji M, Shimotohno K. Cyclosporin A suppresses replication of hepatitis C virus genome in cultured hepatocytes. Hepatology. 2003;38(5):1282-8. Epub 2003/10/28. doi: 10.1053/jhep.2003.50449. PubMed PMID: 14578868.
