# Supplementary material for: Should a viral genome stay in the host cell or leave? A quantitative dynamics study of how hepatitis C virus deals with this dilemma
Source: PLoS Biol. 2020 Jul 30;18(7):e3000562. doi: 10.1371/journal.pbio.3000562 (PMC7392214; doi:10.1371/journal.pbio.3000562)
Supplement: S1 Text — ODE, ordinary differential equation; PDE, partial differential equation. (DOCX) [file pbio.3000562.s016.docx]

**S1 Text: Transformation to a system of ODEs from a PDE multiscale model**

We here introduce an original multiscale model formulated from partial differential equations (PDEs) that couples intra- and inter-cellular virus dynamics for analyzing multiscale experimental data of HCV JFH-1 and Jc1-n in cell culture (cf. [1,2]):

$$\frac{dT\left( t \right)}{dt}=gT\left( t \right)\left( 1-\frac{T\left( t \right)+I\left( t \right)}{K} \right)-\beta T\left( t \right)V_{I}\left( t \right), \left( S1 \right)$$

$$\frac{dI\left( t \right)}{dt}=gI\left( t \right)\left( 1-\frac{T\left( t \right)+I\left( t \right)}{K} \right)+\beta T\left( t \right)V_{I}\left( t \right), \left( S2 \right)$$

$$\left( \frac{\partial}{\partial t}+\frac{\partial}{\partial a} \right)r\left( t,a \right)=kr\left( t,a \right)-\left( \mu+\rho\right)r\left( t,a \right), \left( S3 \right)$$

$$\frac{dV_{I}\left( t \right)}{dt}=f\rho\int_{0}^{\infty} r\left( t,a \right)da-rV_{I}\left( t \right)-cV_{I}\left( t \right), \left( S4 \right)$$

$$\frac{dV_{NI}\left( t \right)}{dt}=\left( 1-f \right)\rho\int_{0}^{\infty} r\left( t,a \right)da+rV_{NI}\left( t \right)-cV_{NI}\left( t \right), \left( S5 \right)$$

with the boundary condition of $r\left( t,0 \right)=\beta T\left( t \right)V_{I}\left( t \right)$. The intercellular variables $T\left( t \right)$ and $I\left( t \right)$ represent the number of uninfected and infected target cells, respectively, and $V_{I}\left( t \right)$ and $V_{NI}\left( t \right)$ denote the amounts of infectious and non-infectious viruses, respectively [3,4] (**Fig 1C**). In addition, we defined the intracellular viral RNA in total infected cells with infection age $a$ as $r\left( t,a \right)$ (**Fig 1B**). The definition of an age-structured population model is found in [5]. The target cells are assumed to grow in a logistic manner as described in Eq. (S11) in **S4 Text**, and to be infected by viruses at rate $\beta$. We considered that intracellular viral RNA replicates at rate $k$, degrades at rate $\mu$, and is exported out of cells at rate $\rho$. That is, infected cells produce viral RNA assembled with viral proteins as virus particles, of which a fraction $f$ are infectious and $1-f$ are non-infectious, and the progeny viruses are cleared at rate $c$. Infectious virions lose infectivity at rate $r$ [3,4].

As we recently reported in [6,7], the multiscale PDE model can also be transformed into a mathematically identical set of ordinary differential equations (ODEs), Eqs. (2–6), as follows. We defined the total amount of intracellular viral RNA, denoted by $A\left( t \right)$, which is calculated by integrating the age distribution over the infection age $a$:

$$A\left( t \right)=\int_{0}^{\infty} r\left( t,a \right)da. \left( S6 \right)$$

Note that $r\left( t,a \right)=0$ if $a>t$. Differentiating $A\left( t \right)$ with respect to time $t$, we obtained the following differential equation:

$$\frac{dA\left( t \right)}{dt}=\int_{0}^{\infty} \frac{\partial}{\partial t}r\left( t,a \right)da. \left( S7 \right)$$

From Eq. (S4), we have:

$$\frac{\partial}{\partial t}r\left( t,a \right)=-\frac{\partial}{\partial a}r\left( t,a \right)+\left( k-\mu-\rho\right)r\left( t,a \right). \left( S8 \right)$$

Then, the differentiation of $A\left( t \right)$ is evaluated as follows:

$$\begin{aligned} \frac{dA\left( t \right)}{dt}=&r\left( t,t \right)+\int_{0}^{t} \frac{\partial}{\partial t}r\left( t,a \right)da \\ =&r\left( t,t \right)+\int_{0}^{t} \left\{ -\frac{\partial}{\partial a}r\left( t,a \right)+\left( k-\mu-\rho\right)r\left( t,a \right) \right\}da \\ =&r\left( t,t \right)-\left[ r\left( t,a \right) \right]_{0}^{t}+\left( k-\mu-\rho\right)A\left( t \right) \\ =&r\left( t, 0 \right)+\left( k-\mu-\rho\right)A\left( t \right). \end{aligned} \left( S9 \right)$$

Because $r\left( t,0 \right)=\beta T\left( t \right)V_{I}\left( t \right)$, the following differential equation is obtained:

$$\frac{dA\left( t \right)}{dt}=\beta T\left( t \right)V_{I}\left( t \right)+\left( k-\mu-\rho\right)A\left( t \right).$$

Taken together, the multiscale PDE model is transformed into the following equivalent system of ODEs:

$$\frac{dT\left( t \right)}{dt}=gT\left( t \right)\left( 1-\frac{T\left( t \right)+I\left( t \right)}{K} \right)-\beta T\left( t \right)V_{I}\left( t \right), \left( S10 \right)$$

$$\frac{dI\left( t \right)}{dt}=gI\left( t \right)\left( 1-\frac{T\left( t \right)+I\left( t \right)}{K} \right)+\beta T\left( t \right)V_{I}\left( t \right), \left( S11 \right)$$

$$\frac{dA\left( t \right)}{dt}=\beta T\left( t \right)V_{I}\left( t \right)+\left( k-\mu-\rho\right)A\left( t \right), \left( S12 \right)$$

$$\frac{dV_{I}\left( t \right)}{dt}=f\rho A\left( t \right)-rV_{I}\left( t \right)-cV_{I}\left( t \right), \left( S13 \right)$$

$$\frac{dV_{NI}\left( t \right)}{dt}=\left( 1-f \right)\rho A\left( t \right)+rV_{NI}\left( t \right)-cV_{NI}\left( t \right). \left( S14 \right)$$

In our experiments, the viral titer was measured both as the total numbers of extracellular virions, expressed as RNA copies/well, and as extracellular infectious virions, expressed as ffu/well (proportional to the concentration of infectious virions). To analyze our cell culture experimental datasets (**Fig 2A**), the time-course of the number of uninfected and infected cells, the extracellular viral titer consisting of RNA copies/well and ffu/well, and the intracellular viral RNA copies/well for HCV JFH-1 and Jc1-n, we further transformed Eqs. (S6–10) into the following scaled model (used in our main text) as described previously [3,4]:

$$\frac{dT\left( t \right)}{dt}=gT\left( t \right)\left( 1-\frac{T\left( t \right)+I\left( t \right)}{K} \right)-\beta_{\theta}T\left( t \right)V_{\theta}\left( t \right), \left( 2 \right)$$

$$\frac{dI\left( t \right)}{dt}=gI\left( t \right)\left( 1-\frac{T\left( t \right)+I\left( t \right)}{K} \right)+\beta_{\theta}T\left( t \right)V_{\theta}\left( t \right), \left( 3 \right)$$

$$\frac{dA\left( t \right)}{dt}=\beta_{\theta}T\left( t \right)V_{\theta}\left( t \right)+\left( k-\mu-\rho\right)A\left( t \right), \left( 4 \right)$$

$$\frac{dV_{\theta}\left( t \right)}{dt}=f_{\theta}\rho A\left( t \right)-rV_{\theta}\left( t \right)-cV_{\theta}\left( t \right), \left( 5 \right)$$

$$\frac{dV\left( t \right)}{dt}=\rho A\left( t \right)-cV\left( t \right). \left( 6 \right)$$

Here $V\left( t \right)=V_{I}\left( t \right)+V_{NI}\left( t \right)$ is the total amount of extracellular viral RNA (copies/well), $V_{\theta}\left( t \right)=\alpha V_{I}\left( t \right)$ is the infectious extracellular viral load expressed in ffu/well, and $\alpha$ is the conversion factor from infectious viral RNA copies/well to ffu/well. The parameters $\beta_{\theta}=\beta/\alpha$ and $f_{\theta}=\alpha f$ are the converted infection rate constant and fraction of infectious virus, respectively. Note that the viral loads (RNA copies and ffu) must be decreased in our model at rate $c=c_{w}+c_{RNA}$ where $c_{w}$ and $c_{RNA}$ correspond to the reductions due to the daily change of medium and viral RNA degradation, respectively. In separate experiments, we directly estimated $c_{w}$, $c_{RNA}$, $\mu+\rho$ and $r$ for both HCV JFH-1 and Jc1-n in Panels A–C, **S1 Fig**. These estimated parameter values are listed in **Table 1**.

**Supplementary References**

1. Guedj J, Dahari H, Rong LB, Sansone ND, Nettles RE, Cotler SJ, et al. Modeling shows that the NS5A inhibitor daclatasvir has two modes of action and yields a shorter estimate of the hepatitis C virus half-life. Proceedings of the National Academy of Sciences of the United States of America. 2013;110(10):3991-6. doi: 10.1073/pnas.1203110110. PubMed PMID: WOS:000316377400069.

2. Rong LB, Guedj J, Dahari H, Coffield DJ, Levi M, Smith P, et al. Analysis of Hepatitis C Virus Decline during Treatment with the Protease Inhibitor Danoprevir Using a Multiscale Model. Plos Computational Biology. 2013;9(3):e1002959. doi: ARTN e1002959

10.1371/journal.pcbi.1002959. PubMed PMID: WOS:000316864200031.

3. Iwami S, Holder BP, Beauchemin CA, Morita S, Tada T, Sato K, et al. Quantification system for the viral dynamics of a highly pathogenic simian/human immunodeficiency virus based on an in vitro experiment and a mathematical model. Retrovirology. 2012;9(1):18. Epub 2012/03/01. doi: 10.1186/1742-4690-9-18. PubMed PMID: 22364292; PubMed Central PMCID: PMCPMC3305505.

4. Iwanami S, Kakizoe Y, Morita S, Miura T, Nakaoka S, Iwami S. A highly pathogenic simian/human immunodeficiency virus effectively produces infectious virions compared with a less pathogenic virus in cell culture. Theor Biol Med Model. 2017;14(1):9. Epub 2017/04/23. doi: 10.1186/s12976-017-0055-8. PubMed PMID: 28431573; PubMed Central PMCID: PMCPMC5401468.

5. Inaba H. Age-structured population dynamics in demography and epidemiology: Springer; 2017.

6. Kitagawa K, Nakaoka S, Asai Y, Watashi K, Iwami S. A PDE multiscale model of hepatitis C virus infection can be transformed to a system of ODEs. J Theor Biol. 2018;448:80-5. Epub 2018/04/11. doi: 10.1016/j.jtbi.2018.04.006. PubMed PMID: 29634960.

7. Kitagawa K, Kuniya T, Nakaoka S, Asai Y, Watashi K, Iwami S. Mathematical Analysis of a Transformed ODE from a PDE Multiscale Model of Hepatitis C Virus Infection. Bull Math Biol. 2019;81(5):1427-41. Epub 2019/01/16. doi: 10.1007/s11538-018-00564-y. PubMed PMID: 30644067.
