# Supplementary material for: Should a viral genome stay in the host cell or leave? A quantitative dynamics study of how hepatitis C virus deals with this dilemma
Source: PLoS Biol. 2020 Jul 30;18(7):e3000562. doi: 10.1371/journal.pbio.3000562 (PMC7392214; doi:10.1371/journal.pbio.3000562)
Supplement: S2 Text — (DOCX) [file pbio.3000562.s017.docx]

**S2 Text: Validation of the difference in virus entry and production between JFH-1 and Jc1-n**

The difference in virus entry between JFH-1 and Jc1-n was validated using the trans-complemented HCV particle (HCVtcp) assay as described [1] (see **S2 Protocol**). The HCVtcp assay can evaluate the process of HCV envelope-mediated entry followed by RNA replication by detecting luciferase activity at 72–96 h as previously described [1]. The HCVtcp assay used a different construct for the HCV envelope derived from either JFH-1 (JFH-1) or J6 (Jc-1n) strain, enabling us to compare the entry of JFH-1 and Jc-1n, as the replication activity of JFH-1 and Jc-1n was the same. As shown in **S3A** **Fig**, luciferase activity driven by the infection of J6 (Jc-1n) was approximately 1.65-fold higher than that driven by JFH-1 infection. Thus, the higher entry activity of Jc-1n than JFH-1 was validated by this independent experiment.

In addition, differences in virus production between Jc1-n and JFH-1 were validated using a single-round HCV production assay as described [2] (see **S3 Protocol**). As shown in **S3B** **Fig**, levels of Jc-1n RNA released from producer cells were remarkably higher than levels of JFH-1 RNA, which was consistent with our conclusion regarding the more rapid release of Jc-1n progeny viruses compared with JFH-1.

Early replication activity was examined by detecting the expression of HCV core and NS5A proteins in a single-round HCV production assay at early time points post-transfection with RNA derived from either JFH-1 or Jc1-n (16, 20, 25, and 30 h post-transfection). As shown in **S3C Fig**, HCV protein production at early time points did not show a clear difference between JFH-1 and Jc1-n.

As a representative host response against HCV infection, IFN induction/response was examined in Huh7.5.1 cells infected with either JFH-1 or Jc1-n. Expression of representative IFN-stimulated genes, MxA and ISG56, as well as HCV core, NS5A, and actin proteins were detected by immunoblotting. IFN-α treatment was used as a positive control for inducing these ISGs. As shown in **S3D** **Fig**, ISGs were not induced by infection with either JFH-1 or Jc1-n in Huh7.5.1 cells, as reported previously [3].

A recent report showed that HCV particle assembly is modulated by the N6-methyladenosine modification status of HCV RNA and is regulated by m6A methyltransferases (METTLs) and m6A-binding YTHDF proteins [4]. We assessed the expression of METTL14 and YTHDF1, 2, and 3, which regulate N6-methyladenosine modification and HCV particle assembly, in JFH-1- or Jc1-n-infected cells at different infection ages (days 3, 5, and 7). As shown in **S3E** **Fig**, expression of these proteins was unchanged by infection age or by infection with any HCV strain.

**Supplementary References**

1. Suzuki R, Saito K, Kato T, Shirakura M, Akazawa D, Ishii K, et al. Trans-complemented hepatitis C virus particles as a versatile tool for study of virus assembly and infection. Virology. 2012;432(1):29-38. Epub 2012/06/26. doi: 10.1016/j.virol.2012.05.033. PubMed PMID: 22727832.

2. Nakajima S, Watashi K, Ohashi H, Kamisuki S, Izaguirre-Carbonell J, Kwon AT, et al. Fungus-Derived Neoechinulin B as a Novel Antagonist of Liver X Receptor, Identified by Chemical Genetics Using a Hepatitis C Virus Cell Culture System. J Virol. 2016;90(20):9058-74. Epub 2016/08/05. doi: 10.1128/JVI.00856-16. PubMed PMID: 27489280; PubMed Central PMCID: PMCPMC5044839.

3. Sumpter R, Jr., Loo YM, Foy E, Li K, Yoneyama M, Fujita T, et al. Regulating intracellular antiviral defense and permissiveness to hepatitis C virus RNA replication through a cellular RNA helicase, RIG-I. J Virol. 2005;79(5):2689-99. Epub 2005/02/15. doi: 10.1128/JVI.79.5.2689-2699.2005. PubMed PMID: 15708988; PubMed Central PMCID: PMCPMC548482.

4. Gokhale NS, McIntyre ABR, McFadden MJ, Roder AE, Kennedy EM, Gandara JA, et al. N6-Methyladenosine in Flaviviridae Viral RNA Genomes Regulates Infection. Cell Host Microbe. 2016;20(5):654-65. Epub 2016/10/25. doi: 10.1016/j.chom.2016.09.015. PubMed PMID: 27773535; PubMed Central PMCID: PMCPMC5123813.
