# Supplementary material for: Should a viral genome stay in the host cell or leave? A quantitative dynamics study of how hepatitis C virus deals with this dilemma
Source: PLoS Biol. 2020 Jul 30;18(7):e3000562. doi: 10.1371/journal.pbio.3000562 (PMC7392214; doi:10.1371/journal.pbio.3000562)
Supplement: S3 Text — (DOCX) [file pbio.3000562.s018.docx]

**S3 Text: Derivation of the Malthusian parameter**

The initial growth of intracellular viral RNA depends on the sub-system of Eqs. (2–6):

$$\begin{aligned} \frac{dA\left( t \right)}{dt}&=\beta_{\theta}T\left( t \right)V_{\theta}\left( t \right)+\left( k-\mu-\rho\right)A\left( t \right), \left( 4 \right) \\ \frac{dV_{\theta}\left( t \right)}{dt}&=f_{\theta}\rho A\left( t \right)-\left( r+c \right)V_{\theta}\left( t \right). \left( 5 \right) \end{aligned}$$

We assumed $T\left( t \right)=K, I\left( t \right)=0$ at the beginning of infection and thus linearized the system:

$$\frac{d}{dt}\left( \begin{aligned} V_{\theta} \\ A \end{aligned} \right)=\left( \begin{matrix} -\left( r+c \right) & f_{\theta}\rho\\ \beta_{\theta}K & k-\mu-\rho\end{matrix} \right)\left( \begin{aligned} V_{\theta} \\ A \end{aligned} \right). \left( S15 \right)$$

Then, the Malthusian parameter is calculated as the maximum eigenvalue of the matrix:

$$M=\frac{k-\mu-\rho-r-c+\sqrt{\left( k-\mu-\rho+r+c \right)^{2}+4\beta_{\theta}Kf_{\theta}\rho}}{2}. \left( 7 \right)$$
