# Supplementary material for: Should a viral genome stay in the host cell or leave? A quantitative dynamics study of how hepatitis C virus deals with this dilemma
Source: PLoS Biol. 2020 Jul 30;18(7):e3000562. doi: 10.1371/journal.pbio.3000562 (PMC7392214; doi:10.1371/journal.pbio.3000562)
Supplement: S4 Text — (DOCX) [file pbio.3000562.s019.docx]

**S4 Text: Quantitation of cell growth**

We estimated the growth kinetics of Huh7.5.1 cells using the following mathematical model:

$$\frac{d\left( T\left( t \right)+I\left( t \right) \right)}{dt}=g\left( T\left( t \right)+I\left( t \right) \right)\left( 1-\frac{T\left( t \right)+I\left( t \right)}{K} \right), \left( S16 \right)$$

where the variables$T\left( t \right)$ and $I\left( t \right)$ represent the numbers of uninfected and infected cells at time $t$, respectively, and the parameters $g$ and $K$ represent the growth rate and the carrying capacity of the cell culture well, respectively. Note that both HCV JFH-1 and Jc1-n infections do not affect growth kinetics because of minimal cytotoxicity [1,2]. Nonlinear least-squares regression (FindMinimum package of Mathematica9.0) was performed to fit Eq. (S11) to the time-course numbers of Huh7.5.1 cells. The fitted parameter values are listed in **Table 1** and the model behavior using these best-fit parameter estimates is presented together with the data in **S1D** **Fig**.

**Supplementary References**

1. Lindenbach BD, Evans MJ, Syder AJ, Wolk B, Tellinghuisen TL, Liu CC, et al. Complete replication of hepatitis C virus in cell culture. Science. 2005;309(5734):623-6. Epub 2005/06/11. doi: 10.1126/science.1114016. PubMed PMID: 15947137.

2. Pietschmann T, Kaul A, Koutsoudakis G, Shavinskaya A, Kallis S, Steinmann E, et al. Construction and characterization of infectious intragenotypic and intergenotypic hepatitis C virus chimeras. Proceedings of the National Academy of Sciences of the United States of America. 2006;103(19):7408-13. Epub 2006/05/03. doi: 10.1073/pnas.0504877103. PubMed PMID: 16651538; PubMed Central PMCID: PMCPMC1455439.
