# Supplementary material for: Should a viral genome stay in the host cell or leave? A quantitative dynamics study of how hepatitis C virus deals with this dilemma
Source: PLoS Biol. 2020 Jul 30;18(7):e3000562. doi: 10.1371/journal.pbio.3000562 (PMC7392214; doi:10.1371/journal.pbio.3000562)
Supplement: S5 Text — (DOCX) [file pbio.3000562.s020.docx]

**S5 Text: Quantitation of clearance rate due to washing**

We assumed that a difference in the logarithmic concentration of extracellular viral RNA before and after changing medium (washing) once a day corresponded to the rate of continuous exponential decay, and calculated the following values for JFH-1 and Jc1-n:

$$c_{w}=\log\frac{V_{b}}{V_{a}}, \left( S17 \right)$$

where the variables $V_{b}$ and $V_{a}$ represent the concentration of extracellular viral RNA before and after washing, respectively. The calculated values are listed in **Table 1**. The data used in this calculation were obtained using the assay described in **S1 Protocol**.
